# Shared Genetic Basis and Causality Between Epilepsy and Psychiatric Disorders: Evidence From a Comprehensive Genetic Analysis

**DOI:** 10.1002/brb3.71267

**Published:** 2026-02-24

**Authors:** Xia Feng, Huan Yao, Gui Xiao

**Affiliations:** ^1^ Department of Sleep Medicine the Second People's Hospital of Guizhou Province Guiyang China; ^2^ Department of Nursing Guizhou Provincial People's Hospital Guiyang China; ^3^ Department of Nursing Jiangxi University of Chinese Medicine Nanchang China

**Keywords:** epilepsy, Mendelian randomization, pleiotropy, psychiatric disorders, shared genetics

## Abstract

**Background:**

Growing evidence suggests that epilepsy and psychiatric disorders may share common genetic underpinnings, yet the precise etiological relationship remains unclear. Psychiatric comorbidities affect approximately 30% of individuals with epilepsy, a rate markedly higher than in the general population, with depression (∼23%) and anxiety (∼20%) being the most prevalent. This high comorbidity burden not only worsens prognosis but also complicates management, underscoring the need for genetic insights into their relationship. To address this gap, we aimed to systematically evaluate the genetic correlation, pleiotropy, and potential causal associations between epilepsy and 14 major psychiatric disorders.

**Methods:**

We analyzed N million single‐nucleotide polymorphisms (SNPs) from genome‐wide association study (GWAS) summary statistics of epilepsy and 14 psychiatric disorders. These GWAS data were obtained from large international consortia, primarily comprising individuals of European ancestry. First, we assessed the genetic correlation between epilepsy and 14 psychiatric disorders using Linkage Disequilibrium Score Regression (LDSC). Second, we used Pleiotropic Analysis under the Composite Null Hypothesis (PLACO) to identify pleiotropic loci at the SNP level. Summary genotype‐phenotype association statistics were used, excluding SNPs with extreme Z^2^ values (>80), and testing for pleiotropy with the Inverse‐Variance Weighted (IVW) method. For gene‐level pleiotropy, we conducted genome annotation multi‐marker analysis (MAGMA v.1.07b). This analysis aggregated SNP‐level associations into gene‐level signals, focusing on 18,563 protein‐coding genes on autosomes. Gene positions were obtained from the Ensembl build (GRCh37) and 1000G EUR data. Functional mapping and annotation of pleiotropic loci were performed using Functional Mapping and Annotation (FUMA). Finally, the bidirectional Mendelian randomization (MR) method was used to investigate causal correlations between epilepsy and 14 psychiatric disorders.

**Results:**

We identified a significant genetic link between epilepsy and attention deficit and hyperactivity disorder (ADHD) (*r_g_* = 0.252, *P* < 0.001), between epilepsy and schizophrenia (SCZ) (*r_g_* = ‐0.060, *p* = 0.003), and between epilepsy and major depressive disorder (MDD) (*r_g_* = 0.167, *p* = 0.014). The genetic correlation between epilepsy and ADHD, epilepsy, and SCZ passed the Bonferroni correction (0.05/14 = 0.0035). Nine shared genetic loci and six pleiotropic genes, including SCN1A, PGBD1, ZKSCAN3, ZKSCAN4, VRK2, and ZSCAN23, have been identified between epilepsy and psychiatric disorders. Furthermore, these loci and genes mainly involve the MAPK signaling pathway. MR analysis showed ADHD (OR = 1.097, 95% CI: 1.019‐1.180, *p* = 0.014) and MDD (OR = 1.277, 95% CI 1.114‐1.463, *p* = 0.000) are the risk factors for epilepsy. BIP is the protecting factor against epilepsy (OR = 0.930, 95% CI: 0.878‐0.986, *p* = 0.014). The causality between MDD and epilepsy passed the Bonferroni correction (0.05/14 = 0.0035).

**Conclusions:**

SCZ, ADHD, MDD and epilepsy may share a common etiology, respectively. These etiologies may be related to precise molecular mechanisms, leading to overlapping pathological physiology and clinical features. These findings may offer insights into treatment trials.

## Graphical abstract

We identified shared genetic architecture and bidirectional causal associations between epilepsy and major psychiatric disorders, highlighting MAPK signaling as a potential convergent pathway.

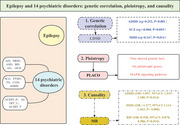

## 1 Introduction

Epilepsy is one of the most prevalent neurological disorders, affecting over 70 million people worldwide (Thijs et al. 2019). Following the International League Against Epilepsy (ILAE), epilepsy is defined as a brain disease characterized by an enduring predisposition to generate epileptic seizures and their neurobiological, cognitive, psychological, and social consequences (Riney et al. 2022). Unfortunately, despite the increasing availability of drugs, improvements in outcomes have only been marginal. Anti‐seizure medications are the primary treatment modalities for most patients with epilepsy. Globally, more than 25 medications are available; however, the current drugs are effective in only approximately 66% of individuals in high‐income countries (Duncan et al. 2006). In addition, surveys from 2013 and 2015 in the USA revealed that more than half of those taking epilepsy medications still experienced seizures (Tian et al. 2018). Therefore, it is essential to investigate the pathogenesis of epilepsy and develop novel therapeutic strategies to alleviate this substantial socioeconomic burden.

Most patients with epilepsy have comorbidities, and psychiatric disorders are particularly common, affecting about one in three over their lifetime (Campbell et al. 2020). Mood and anxiety disorders are the most frequent, with prevalence rates up to 30%–35% in both adults and children (Gaitatzis et al. 2004), while ADHD is especially common in pediatric epilepsy (13%–50%), with many cases persisting into adulthood (Dunn et al. 2003; Thapar and Cooper 2016). These comorbidities impose substantial social and economic burdens by exacerbating stigma, impairing quality of life, and increasing healthcare utilization; for instance, depression and anxiety affect roughly one in five patients and contribute to unemployment and high family and healthcare costs (Mohanannair Geethadevi et al. 2025). Given their high prevalence and significant impact, it is essential to elucidate their underlying mechanisms. Evidence suggests that epilepsy and psychiatric disorders share common genetic etiologies, which encompass inherited polygenic variation, shared susceptibility loci, and pleiotropic molecular mechanisms contributing to disease vulnerability. However, these genetic links remain poorly understood (Pisani et al. 2023).

Accumulating biological evidence suggests that epilepsy and several psychiatric disorders may involve overlapping neurobiological pathways that contribute to shared genetic liability. Studies have shown that altered neuronal excitability and ion‐channel dysfunction—particularly involving genes such as SCN1A and CACNA1C‐play key roles in both epileptogenesis and psychiatric phenotypes (Bozarth et al. 2018). Disruptions in GABAergic and glutamatergic neurotransmission have similarly been implicated in seizure susceptibility as well as mood and psychotic disorders (Witkin et al. 2024). In addition, neuroinflammatory mechanisms, including cytokine‐mediated alterations in neuronal activity, have been associated with both depression and epilepsy (Hollis and Lukens 2025). Dysregulated intracellular signaling pathways, such as MAPK/ERK signaling, have further been linked to neuronal plasticity and symptom development across multiple neuropsychiatric conditions (Albert‐Gascó et al. 2020). Together, these findings suggest that shared molecular mechanisms may underlie the frequent co‐occurrence of epilepsy and psychiatric disorders and offer a biological foundation for investigating shared genetic risk.

A systematic analysis is therefore needed to identify pleiotropic risk variants and clarify potential shared pathways. Understanding the links between epilepsy and psychiatric disorders is essential. Shared genetic risk factors or pathways may explain their frequent co‐occurrence, enabling better prediction and monitoring of psychiatric complications and suggesting therapeutic targets for both neurological and psychiatric aspects. Epilepsy itself is a highly polygenic disease driven by genetic variation. A new method called polygenic analysis has been developed based on the polygenic null hypothesis (PLACO) to identify genetic variations that affect two characteristics or disease risks (Ray and Chatterjee 2020). PLACO uses aggregate‐level genotype‐phenotype association statistics (usually genome‐wide association study [GWAS] statistics). Due to the sharing of control groups between studies, PLACO may allow statistical data to be correlated. The PLACO method maintains type I errors and achieves significant gains over simple alternative methods commonly used to test polygenicity (Ray et al. 2021).

However, no studies have systematically examined the genetic correlations, pleiotropy, or causal links between epilepsy and psychiatric disorders. In this study, we adopt the ILAE classifications of seizures and epilepsies/epilepsy syndromes (2017/2022) (Riney et al. 2022). The GWAS phenotype analyzed corresponds to “all epilepsy,” an umbrella category encompassing both focal and generalized forms as aggregated in the source data. On this basis, we comprehensively investigate the relationships between epilepsy and 14 psychiatric disorders using genetic correlation, pleiotropy, functional enrichment, and Mendelian randomization analyses.

## 2 Methods

### 2.1 Ethical Review

All analyses were performed using publicly available data from published literature; therefore, no ethical approval or patient consent was required.

### 2.2 GWAS Summary Statistics

Our study explored the genetic correlation, pleiotropy, and causality between epilepsy (*N* = 44,889, including 15,212 cases and 29,677 controls) (International League Against Epilepsy Consortium on Complex E 2018) and 14 psychiatric disorders. We analyzed publicly available GWAS summary statistics for overall epilepsy, an aggregated phenotype encompassing genetic generalized epilepsy (GGE) and focal epilepsy (FE) as defined in the source studies. Because subtype‐specific summary statistics were not uniformly available, stratified analyses by epilepsy subtype were not performed. The 14 types of psychiatric disorders includes Alzheimer's disease (AD) (71,880 cases and 383,378 controls) (Jansen et al. 2019), attention deficit hyperactivity disorder (ADHD) (19,099 cases and 34,194 controls) (Demontis et al. 2019), autism spectrum disorder (ASD) (18,381 cases and 27,969 controls) (Grove et al. 2019), anorexia nervosa (AN) (3495 cases and 10,982 controls) (Duncan et al. 2017), bipolar disorder (BIP) (41,917 cases and 371,549 controls) (Mullins et al. 2021), major depressive disorder (MDD) (246,363 cases and 561,190 controls) (Howard et al. 2019), obsessive‐compulsive disorder (OCD) (2,688 cases and 7,037 controls) (International Obsessive Compulsive Disorder Foundation Genetics C, Studies OCDCGA 2018), post‐traumatic stress disorder (PTSD) (23,212 cases and 151,447 controls) (Nievergelt et al. 2019), schizophrenia (SCZ) (33,640 cases and 43,456 controls) (Trubetskoy et al. 2022), Tourette's syndrome (TS) (4,819 cases and 9,488 controls) (Yu et al. 2019), and cannabis use (CU) (53,180 cases and 131,585 controls) (Johnson et al. 2020), as well as alcohol use disorder (AUD; with a population of 121,604) (Sanchez‐Roige et al. 2019). The GWAS statistics are summarized in Table 1. A full description of the study design, including diagnostic criteria, sample collection, quality control measures, and imputation methods, is available for each publication. All GWAS protocols were approved by the appropriate institutional review boards or ethics committees.

**TABLE 1:** Summary information of epilepsy and 14 psychiatric disorders used in this study.

Traits	N (case/control)	PMID
Epilepsy	44,889 (15,212/29,677)	30531953
AD	455,258 (71,880/383,378)	30617256
ADHD	53,293 (19,099/34,194)	30478444
AN	14,477 (3495/10,982)	28494655
ASD	46,350 (18,381/27,969)	30804558
BIP	413,466 (41,917/371,549)	34002096
CU	184,765 (53,180/131,585)	33096046
MDD	807,553 (246,363/561,190)	30718901
OCD	9725 (2688/7037)	28761083
PTSD	174,659 (23,212/151,447)	31594949
SCZ	77,096 (33,640/43,456)	35396580
TS	14,307 (4819/9488)	30818990
AUDIT‐T	121,604	30336701
AUDIT‐C	121,604	30336701
AUDIT‐P	121,604	30336701

Abbreviations: AD, Alzheimer's disease; ADHD, attention deficit hyperactivity disorder; AN, anorexia nervosa; ASD, autism spectrum disorder; AUDIT‐C, alcohol use disorder identification test – consumption; AUDIT‐P, alcohol use disorder identification test – alcohol problems; AUDIT‐T, Alcohol Use Disorder Identification Test; BIP, bipolar disorder; CU, cannabis use; MDD, major depressive disorder; OCD, obsessive‐compulsive disorder; PTSD, post‐traumatic stress disorder; SCZ, schizophrenia; TS, Tourette's syndrome.

### 2.3 Genetic Correlation Analysis Using Linkage Disequilibrium Score Regression (LDSC)

**FIGURE 1:**
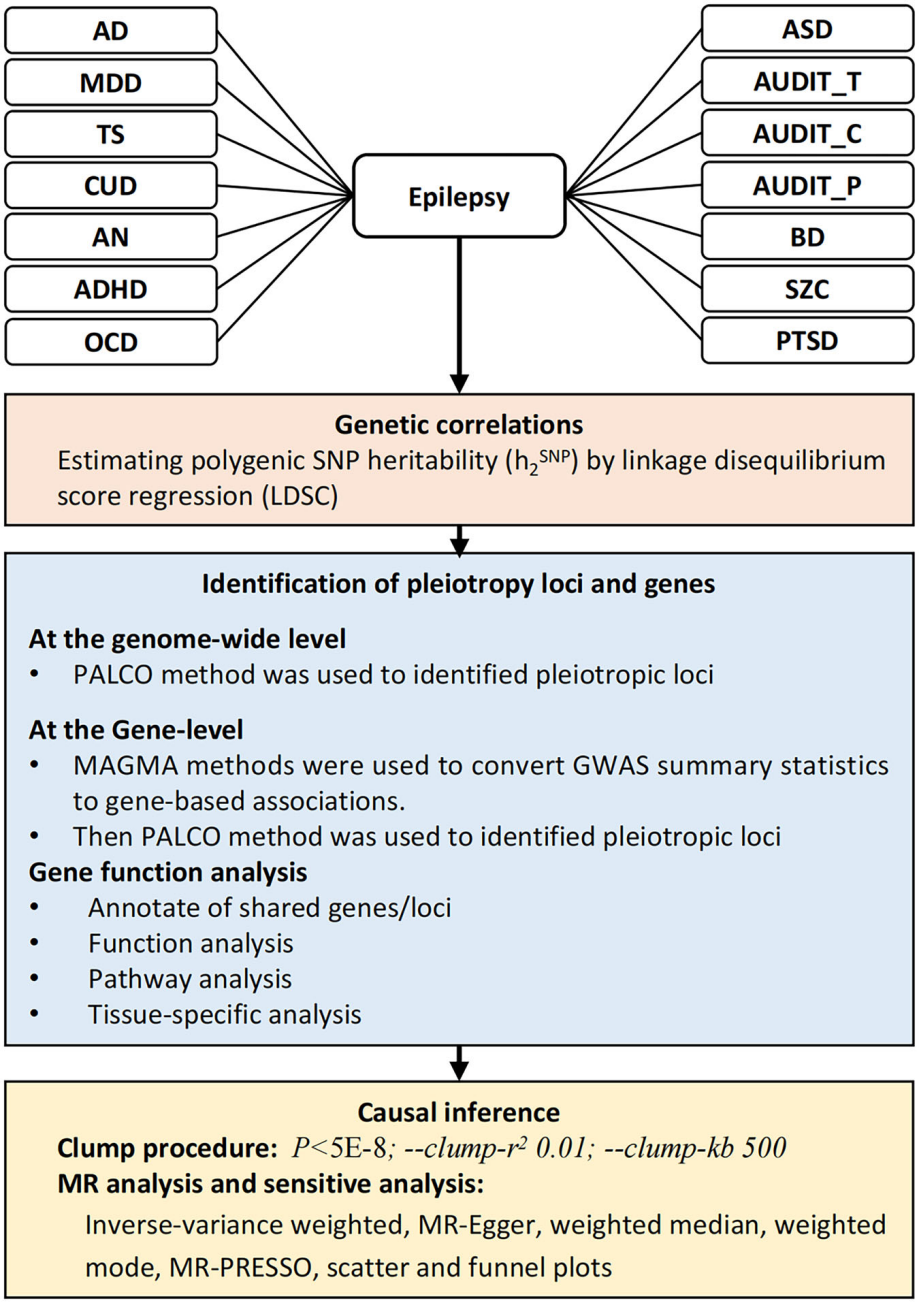
Schematic overview of the analytical workflow.

Figure 1 shows the overall analytical workflow. First, LDSC was used to determine the genetic correlation between the 14 psychiatric disorders and epilepsy (Bulik‐Sullivan et al. 2015). In LDSC, r_g is the genome‐wide standardized covariance of SNP effect sizes (range –1 to +1), interpreted at the polygenic (aggregate) level and not as evidence of individual‐level protection or clinical causation. Two sets of LDSC calculations were performed, based on either the 1000 Genomes Project or HapMap 3 reference data (Genomes Project Consortium et al. 2015). All disorders were analyzed using the European‐only summary statistics, and strict quality control was conducted as follows: (1) Non‐biallelic single nucleotide polymorphisms (SNPs) or ones with strand‐ambiguous alleles were excluded; (2) SNPs without an RS label were excluded; (3) Duplicate SNPs or those SNPs that were not involved in the 1000 Genomes Project or did not match the alleles were removed; (4) SNPs located in the region of the major histocompatibility complex (mainly on chromosome 6:28.5–33.5 Mb) were excluded from the analysis because of the complex LD structure (Bulik‐Sullivan et al. 2015); (5) SNPs with minor allele frequency (MAF) greater than 0.01 were retained. Subsequently, the LDSC performed a weighted linear model by regressing the product of the Z statistics of the two traits on the LD score across all available genetic variants across the whole genome. In theory, even if there is an overlap between two GWASs, the regression slope can still provide an unbiased estimate of genetic correlation. Owing to Bonferroni correction, a *p‐*value less than 0.0035 (0.05/14) was considered significant.

### 2.4 Gene‐Based Pleiotropic Analysis Under PLACO

#### 2.4.1 SNP‐Level

To identify pleiotropic genetic variants between epilepsy and each psychiatric disorder, we employed the PLACO method (Ray and Chatterjee 2020). PLACO tests whether a SNP is associated with both traits versus only one or neither, using a statistic based on the product of GWAS Z‐scores and a null distribution that accounts for sample overlap. This allows detection of variants with dual influences even when their single‐trait effects are modest. Identifying such loci is critical for revealing shared mechanisms and potential therapeutic targets (Zeng et al. 2021). According to previous simulations, a variance‐component‐based mediation analysis suggested that this extension was valid under the composite null hypothesis. PLACO employs two sets of Z‐statistics as inputs to examine one gene at a time and divides pleiotropy into three sub‐null scenarios: (1) H_00_: neither disorder is associated with the gene; (2) H_10_: There is a correlation between the gene and the first disease, but not the second; (3) H_01_: The gene is not related to the first disease but to the second. A possible alternative hypothesis (H_11_) is that this gene is involved in both diseases, indicating its pleiotropic effects. For each variant, the square of the Z‐score was calculated, and the SNPs with extremely high Z^2^ values (>80) were removed. Additionally, considering the potential correlation between psychiatric disorders and epilepsy, a correlation matrix for Z was calculated. The non‐pleiotropy hypothesis was tested using the intersection union test (IUT) method. Therefore, the final *p*‐value of the IUT test was the maximum of the p‐values for H_0_ and H_1_.

#### 2.4.2 Gene‐Level

Annotation multi‐marker analysis (MAGMA v.1.07b) (de Leeuw et al. 2015) detects pleiotropic genes by combining SNP‐level correlations with a single gene‐level correlation signal. Here, MAGMA was used because this method has been shown to be robust and computationally efficient. This MAGMA analysis was restricted to 18,563 protein‐coding genes in the autosomes. This method assigns an adjacent SNP to the same gene by setting an annotation window of 500 kb. Information regarding gene location was extracted from the 1000G EUR data (Sudmant et al. 2015) and the ensemble build (GRCh37) (Cunningham et al. 2015). This methodology was extended to uncover gene‐level pleiotropic correlations. The P‐values of MAGMA and PLACO were also corrected for multiple testing using the Bonferroni correction. Detailed information about PLACO and MAGMA was presented in Supplementary methods.

#### 2.4.3 Gene Function Analysis for Pleiotropic Loci and Genes

In addition, FUMA (https://fuma.ctglab.nl/) was used to analyze the differential expression and gene set enrichment for pleiotropic loci identified by PLACO (Watanabe et al. 2017). MAGMA gene set analysis was conducted to explore the biological functions of the leading SNP (de Leeuw et al. 2015). The identified loci were mapped to nearby genes, and a series of pathway enrichment analyses were conducted using MSigDB (Subramanian et al. 2005) to ultimately determine the functions of the mapped genes. Bonferroni correction was used for multiple comparison corrections for each tested gene set (e.g., Gene Ontology [GO] biological process).

### 2.5 Causal Correlation Between Epilepsy and 14 Psychiatric Disorders Based on MR

MR is a standard method for inferring causal relationships between exposure and outcomes using SNPs associated with exposure as instruments (Sheehan et al. 2008). Bidirectional MR analyses were conducted for 14 patients with psychiatric disorders and epilepsy. Using the PLINK clumping procedure, we selected independent SNPs at a genome‐wide significant level (*P *< 5 × 10^−8^) (Noyce et al. 2017). Clumping selection was conducted by setting an LD threshold of 0.001 and a physical distance of 500 kb. In addition, a reference panel of 503 European ancestries from the 1000 Genomes Project was used to estimate LD.

The explained variance (R^2^) and F‐statistic parameters were calculated to test the validity of the identified inverse variances (IVs). The formulas for F and R^2^ are listed in Table S1. We retained only SNPs with strong association to the exposure, defined by a first‐stage F‐statistic ≥10. This conventional threshold indicates instrument strength sufficient to limit weak‐instrument bias in a finite sample. In two‐sample MR, any residual weak‐instrument bias is expected to attenuate estimates towards the null rather than towards the confounded observational association (Burgess et al. 2017). The main method used to determine the causal effects between 14 psychiatric disorders and epilepsy was inverse variance weighting (IVW) (Bowden et al. 2016). To increase the stability and robustness of the results, additional analyses with different modeling assumptions and advantages were performed (weighted median and weighted mode) (Bowden et al. 2016). There complementary sensitivity analyses were performed to evaluate the robustness of the significant correlation: (1) Cochran's Q statistic, which was calculated to estimate the heterogeneity among SNPs, and (2) the intercept of MR‐Egger regression, which was used to estimate the directional pleiotropy of SNPs (Bowden et al. 2016; Burgess and Thompson 2017), and (3) MR‐PRESSO (pleiotropy residual sum and outlier) methods) were used for sensitivity analyses in MR analysis. MR‐PRESSO is a statistical method used to identify and correct horizontal and vertical biases present in MR analysis (Verbanck et al. 2018).

## 3 Results

### 3.1 Genetic Correlation Between Epilepsy and 14 Psychiatric Disorders Using LDSC

**FIGURE 2:**
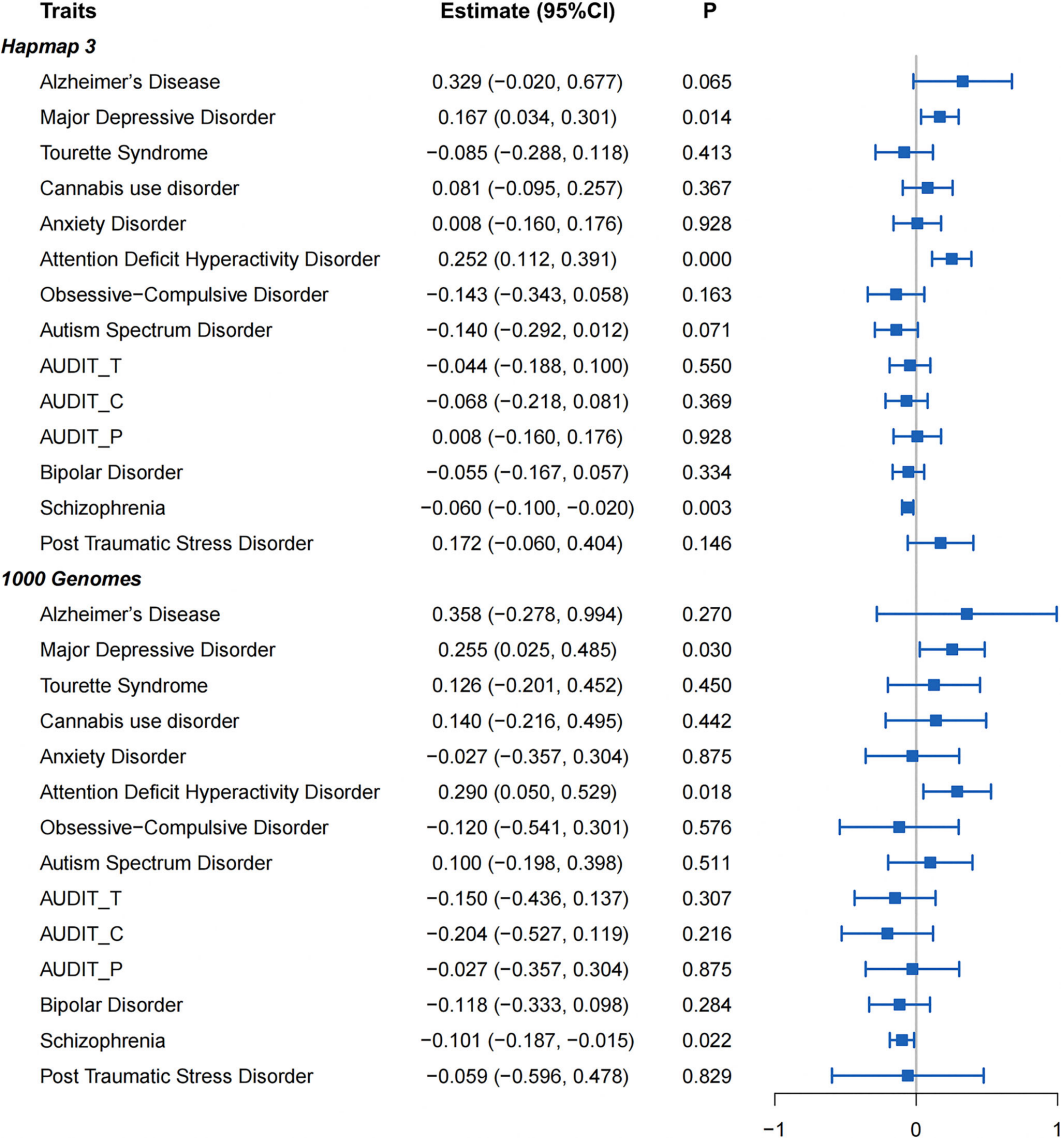
Forest plots of genetic correlation between epilepsy and 14 psychiatric disorders using LDSC.

The LDSC genetic correlation analysis revealed significant genetic correlations between MDD (rg = 0.167, *p* = 0.014), ADHD (rg = 0.252, *p* = 0.0004), SCZ (rg = ‐0.060, *p* = 0.003), and epilepsy (Figure 2 and Table S2). In LDSC, r_g captures the standardized covariance of SNP effect sizes across the genome; thus, a negative value indicates antagonistic polygenic sharing, whereby alleles increasing liability to one trait are, on average, slightly depleted among risk alleles for the other. Given the small magnitude, this signal should be interpreted as subtle, with most genetic architecture remaining trait specific. After correction for multiple testing using the Bonferroni correction, ADHD and SCZ remained significant (0.05/14 = 0.0035). Genetic correlations between epilepsy and ADHD, epilepsy and SCZ suggested that a similar genetic mechanism may link them. Specific genetic loci shared by epilepsy and psychiatric disorders were determined through further analyses.

### 3.2 Pleiotropic Signals Identified by PLACO

**FIGURE 3:**
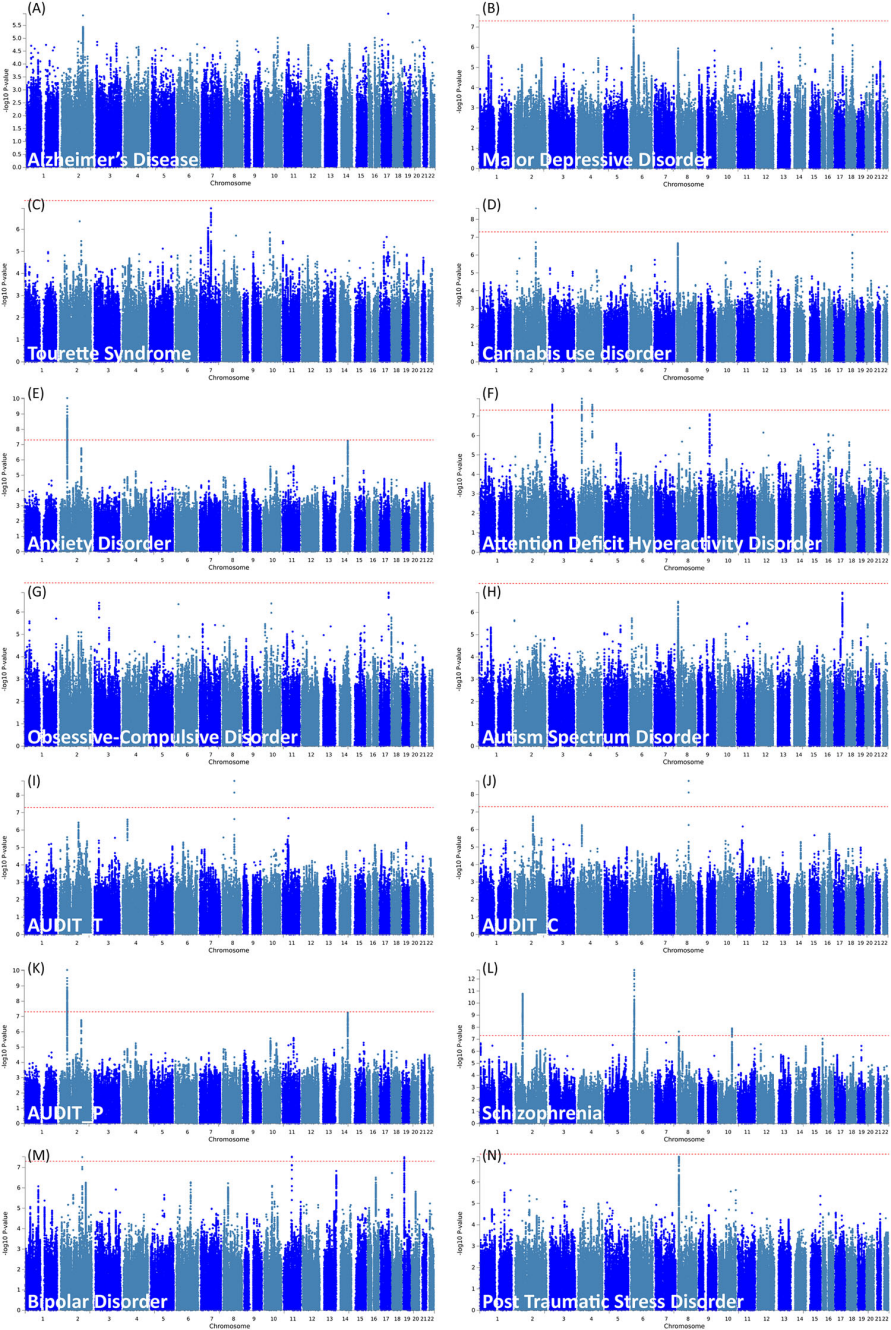
Manhattan plot of pleiotropy between 14 psychiatric disorders and epilepsy. (A) Alzheimer's Disease and epilepsy; (B) Major Depressive Disorder and epilepsy; (C) Tourette Syndrome and epilepsy; (D) Cannabis use disorder and epilepsy; (E) Anxiety Disorder and epilepsy; (F) Attention Deficit Hyperactivity Disorder and epilepsy; (G) Obsessive‐Compulsive Disorder and epilepsy; (H) Autism Spectrum Disorder and epilepsy; (I) AUDIT‐D and epilepsy; (J) AUDIT‐C and epilepsy; (K) AUDIT‐P and epilepsy; (L) Schizophrenia and epilepsy; (M) Bipolar Disorder and epilepsy; (N) Post Traumatic Stress Disorder and epilepsy.

**SNP‐level** Across the 14 pairwise comparisons between epilepsy and each psychiatric disorder, PLACO identified multiple pleiotropic lead SNPs that reached genome‐wide significance (PLACO < 5 × 10^−8^), which could be grouped into nine independent genomic risk loci (Figure 3 and Table 2). At several schizophrenia–epilepsy loci, the estimated effects on the two traits were of opposite sign, in line with the small negative genome‐wide genetic correlation observed in LDSC, whereas loci shared between ADHD and epilepsy generally showed concordant directions of effect, consistent with their positive genetic correlations. By mapping the pleiotropic lead SNPs to nearby protein‐coding genes (Subramanian et al. 2005), we prioritized six candidate pleiotropic genes‐SCN1A, PGBD1, ZKSCAN3, ZKSCAN4, VRK2, and ZSCAN23—for downstream functional and pathway analyses (Table 3).

**TABLE 2:** Pleiotropic loci between 14 psychiatric disorders and epilepsy(*P* < 5E^−8^).

No.	Trait 1	Trait 2	rs	Chromosome	Position	*P*	Nearby Genes
1	AN, AUDIT_P	Epilepsies	rs4671319	2	57950346	9.07244E‐11	LOC647016||LOC100131953
2	BIP, CU	Epilepsies	rs4667876	2	166994996	3.1111E‐08	SCN1A||SCN9A
3	ADHD	Epilepsies	rs4858199	3	20462528	2.58164E‐08	SGOL1||VENTXP7
4	ADHD	Epilepsies	rs9996642	4	31119646	1.28763E‐08	PCDH7
5	ADHD	Epilepsies	rs12500836	4	112230512	2.65813E‐08	LOC391686||LOC132719
6	MDD, SCZ	Epilepsies	rs202906	6	28011652	2.52734E‐08	OR2W2P||OR2B7P
7	AUDIT‐C, AUDIT‐T	Epilepsies	rs4419791	8	93367913	1.67881E‐09	RPS26P10||FLJ46284
8	BIP	Epilepsies	rs489337	11	65854561	2.98454E‐08	PACS1
9	BIP	Epilepsies	rs2742313	19	10799750	3.1378E‐08	ILF3

Abbreviations: ADHD, attention deficit hyperactivity disorder; AN, anorexia nervosa; AUDIT‐C, alcohol use disorder Identification test – consumption; AUDIT‐P, alcohol use disorder Identification Test – alcohol problems; AUDIT‐T, alcohol use disorder Identification test; BIP, bipolar disorder; CU, cannabis use; SCZ, schizophrenia.

**TABLE 3:** Pleiotropic genes between 14 psychiatric disorders and epilepsy(*P* < 0.05/N_genes_).

ID	Trait 1	Trait 2	Genes	*P‐*placo
1	Epilepsies	AD	SCN1A	7.17E‐07
2	Epilepsies	MDD	PGBD1	1.44E‐06
3	Epilepsies	MDD	ZKSCAN3	1.67E‐06
4	Epilepsies	MDD	ZKSCAN4	9.71E‐07
5	Epilepsies	CU	SCN1A	5.66E‐07
6	Epilepsies	AN	SCN1A	9.47E‐07
7	Epilepsies	AUDIT‐P	SCN1A	9.47E‐07
8	Epilepsies	SCZ	SCN1A	9.77E‐07
9	Epilepsies	SCZ	VRK2	1.19E‐08
10	Epilepsies	SCZ	ZKSCAN3	5.31E‐08
11	Epilepsies	SCZ	ZSCAN23	2.31E‐07

Abbreviations: AD, Alzheimer's disease; AN, anorexia nervosa; AUDIT‐P, Alcohol Use Disorder Identification Test – alcohol problems; CU, cannabis use; MDD, major depressive disorder; SCZ, schizophrenia.

**FIGURE 4:**
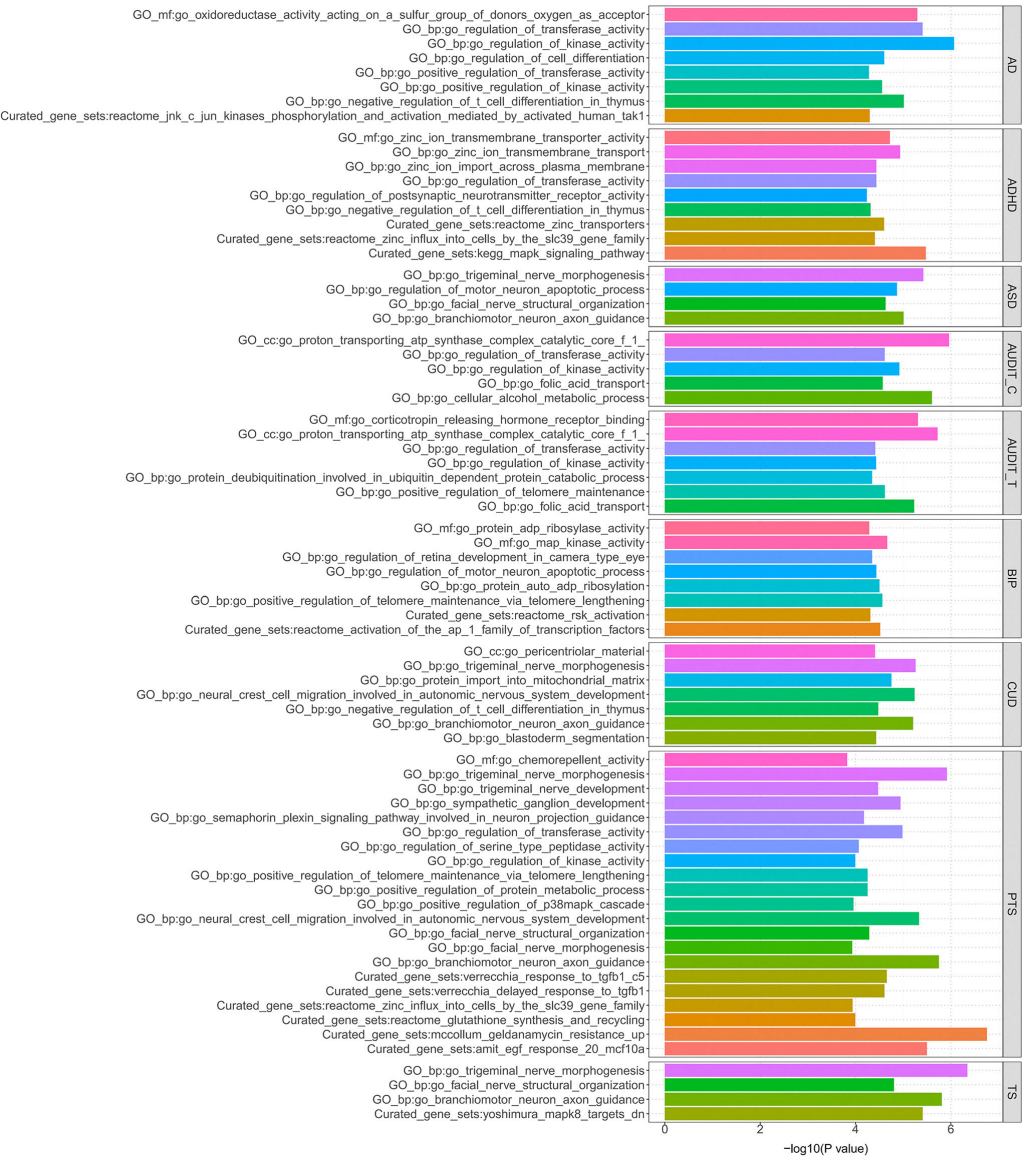
Significant types of pathways in terms of the GO and KEGG enrichment analyses.

Gene‐level MAGMA gene set analysis showed that pleiotropic loci were enriched in different tissues, including the brain and heart (Figure S1). According to the GO enrichment analysis, the pleiotropic loci between epilepsy and 14 psychiatric disorders have high enrichment in biological processes (BP). The main BPs include the regulation of neural development (e.g., cell differentiation and neuron differentiation) and regulation of transferase, kinase, and protein metabolism processes. According to the GO enrichment analysis of cell components, these pleiotropic loci were concentrated in the cytoplasmic perinuclear, neuronal, and proton regions, whereas in the enrichment analysis of molecular function, these pleiotropic loci were enriched in oxidoreductase activity, zinc ion transmembrane transport activity, regulation of postsynaptic neurotransmitter receptor activity, corticotropin‐releasing hormone receptor binding, map kinase activity, and chemorepellent activity. KEGG enrichment analysis showed that these pleiotropic loci were significantly enriched in the mitogen‐activated protein kinase (MAPK) signaling pathway (Figure 4). A pleiotropic analysis and QQ plot of the 14 psychiatric disorders and epilepsy are shown in Figure S2. Moreover, the results showed that in terms of expression levels in GTEx v8 54 tissues, the six pleiotropic genes, including SCN1A, PGBD1, ZKSCAN3, ZKSCAN4, VRK2, and ZSCAN23, were significantly enriched in the brain, skin, liver, and heart tissues (Figures S3 and S4).

### 3.3 Bidirectional Causal Relationships Between Epilepsy and 14 Psychiatric Disorders

**FIGURE 5:**
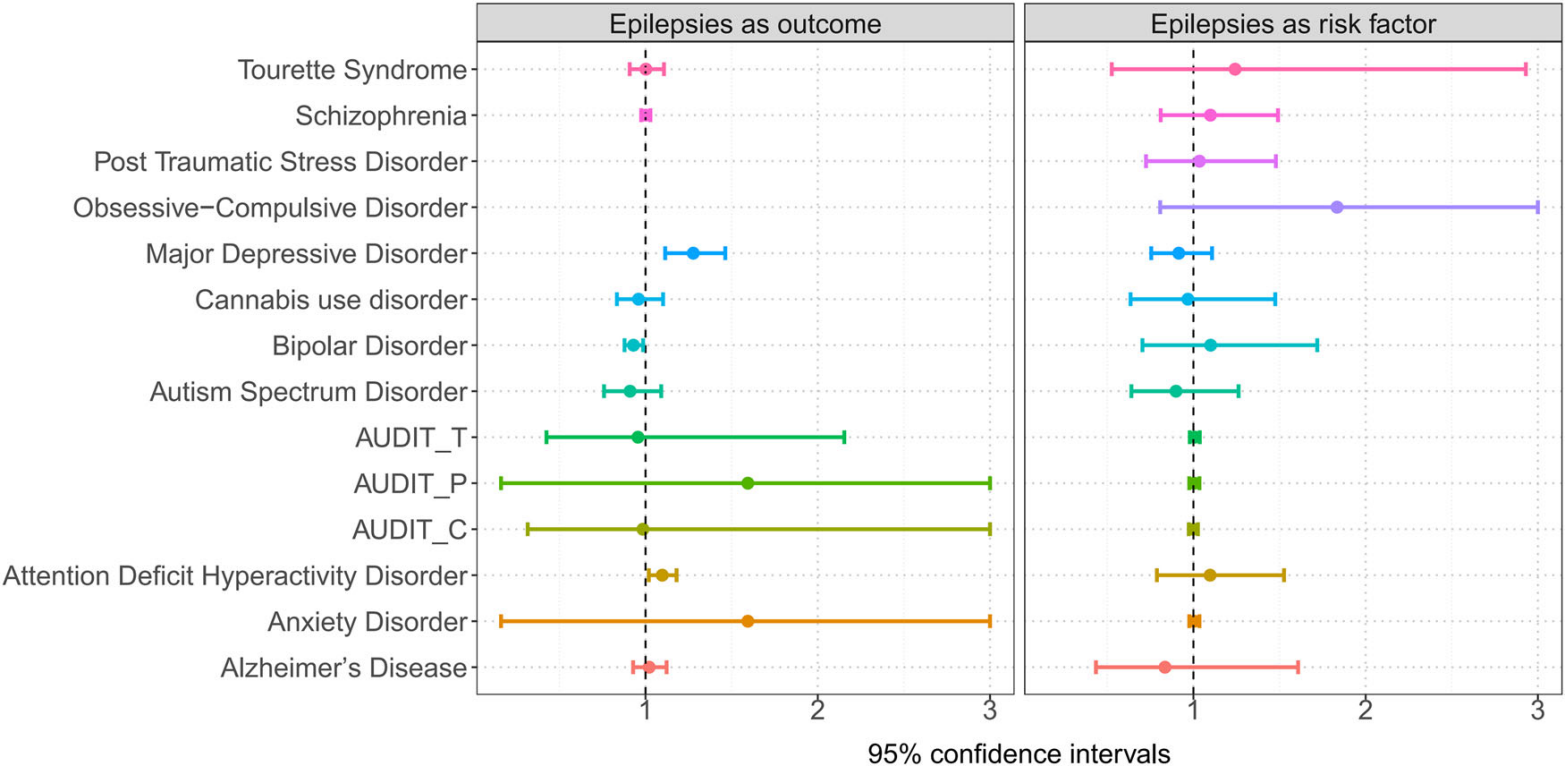
Forest plot of MR results exploring the potential causal relationship between epilepsy and 14 psychiatric disorders.

The number of SNPs significantly associated with psychiatric disorders ranged from one to 64. Moreover, the selected SNPs explained 8.4% of the phenotypic variance in all psychiatric disorders (Tables S3 and S4). Most miniature F‐statistics were greater than 10 (range: 29.64‐37.13), indicating a low possibility of weak instrument bias. According to the IVW method, BIP patients showed a lower risk of epilepsy than normal controls (odds ratio [OR] = 0.930, 95% confidence interval [95%CI]: 0.878–0.986, *p* = 0.014), which is in accordance with the MR‐PRESSO results (OR = 0.930, 95% CI: 0.895–0.968, *p* = 0.005). According to the IVW results, the risk of epilepsy in patients was higher (OR = 1.097, 95% CI: 1.019–1.180, *p* = 0.014), which was in line with the MR‐PRESSO results (OR = 1.097, 95% CI: 1.029–1.169, *p* = 0.037). Similarly, according to the IVW results, the risk of epilepsy in patients was higher (OR = 1.277, 95% CI: 1.114–1.463, *p* = 0.000), which was in line with the MR‐PRESSO results (OR = 1.277, 95%CI: 1.164–1.400, *p* = 0.006) (Figure 4, Figure 5, and Table S4). The causality between MDD and epilepsy passed Bonferroni correction (0.05/14 = 0.0035). The MR‐Egger intercept test indicated that genetic pleiotropy did not affect the basis of BIP (SE = 0.015, *p* = 0.486), ADHD (SE = 0.016, *p* = 0.452), or MDD in epilepsy (SE = 0.026, *p* = 0.964). No heterogeneity was detected in the causality of BIP on epilepsy (IVW.Q = 4.604, *p* = 0.916), ADHD on epilepsy (IVW.Q = 3.794, *p* = 0.579), or MDD on epilepsy (IVW.Q = 1.826, *p* = 0.768) using Cochran's Q test. Meanwhile, the MR‐PRESSO analysis detected no potential instrumental outliers at the nominal significance level of 0.05 (Table S4, Figure S5). In addition, the reverse MR analysis results showed no statistically significant correlation between genetically increased epilepsy risk and 14 types of psychiatric disorders in the European population (Table S5).

## 4 Discussion

To the best of our knowledge, this is the first study to systematically investigate genetic correlations, pleiotropy, and causal relationships between epilepsy and 14 psychiatric disorders. Using large‐scale GWAS statistics, we applied LDSC and found shared genetic overlaps among MDD, ADHD, SCZ, and epilepsy. PLACO identified nine pleiotropic loci and six genes (SCN1A, PGBD1, ZKSCAN3, ZKSCAN4, VRK2, ZSCAN23), with enrichment in pathways such as MAPK signaling and tissues including brain, heart, and skin. Bidirectional MR analysis provided evidence consistent with a potential causal role of ADHD and MDD in increasing epilepsy risk. Conversely, genetic liability to BIP was associated with a reduced risk, suggesting a potential protective relationship. Reverse MR indicated that increased epilepsy risk did not elevate risk for the 14 psychiatric disorders.

Our findings align with observational studies showing comorbidities between epilepsy and SCZ, ADHD, and MDD. Individuals with epilepsy are more likely to develop psychotic symptoms resembling SCZ (Cascella et al. 2009). Moreover, focal impaired‐awareness seizures have been associated with a higher risk of interictal psychosis. It has been suggested that schizophrenia and epilepsy may share pathological features within the medial temporal lobe (Asadi‐Pooya et al. 2017). Population‐based studies, including a Finnish birth cohort (OR = 11.1, 95% CI 4.0‐31.6), confirmed that epilepsy increases the risk of SCZ and SCZ‐like psychoses (Qin et al. 2005; Makikyro et al. 1998). The inverse schizophrenia‐epilepsy r_g observed in our study likely reflects antagonistic sharing of common variant effects rather than a protective mechanism, consistent with MR results indicating horizontal pleiotropy rather than causation (Bulik‐Sullivan et al. 2015). Epilepsy is also closely linked with ADHD. Two large Taiwanese cohort studies demonstrated a bidirectional relationship: epilepsy patients had higher ADHD incidence (adjusted HR = 2.54, 95% CI 2.02‐3.18), while ADHD patients had higher epilepsy incidence (HR = 3.94, 95% CI 2.58‐6.03) (Chou et al. 2013; Socanski et al. 2013). Finally, MDD is another frequent comorbidity, with a meta‐analysis reporting a 21.9% prevalence among epilepsy patients (95% CI 20.8–23.3) (Kim et al. 2018).

The six pleiotropic genes identified between epilepsy and psychiatric disorders have also been implicated in neurological or psychiatric conditions in prior studies, which bolsters their biological plausibility. Notably, one of the shared loci involves the SCN1A gene. Given SCN1A's known role in Dravet syndrome (a genetic epilepsy), its pleiotropic association here may point to mechanisms connecting early neuronal excitability (seizure propensity) with neurodevelopmental outcomes. More broadly, the pleiotropic signals identified in the MAPK pathway provide a concrete biological and clinical validation of our genetic findings. This is exemplified by the case of RASopathies, a well‐defined group of neurodevelopmental disorders caused by pathogenic mutations in the Ras/MAPK pathway. It is clinically established that individuals with RASopathies exhibit a significantly elevated risk of both seizures and neuropsychiatric comorbidities (Escayg et al. 2000; Scheffer and Nabbout 2019; Brunklaus et al. 2022). The convergence between our agnostic genetic analysis–which pinpointed the MAPK pathway–and this established monogenic etiology of epilepsy‐related syndromes strongly enhances the biological plausibility of our results. This example demonstrates that at least a subset of the shared genetic architecture uncovered in our study maps onto specific, high‐effect pathogenic mechanisms with clear clinical consequences, thereby substantiating the relevance of our findings beyond mere statistical association. Beyond SCN1A, several other genes strengthen this interpretation. VRK2 has consistently been linked to SCZ, MDD, BIP, and generalized epilepsy across multiple populations, suggesting its central role in shared genetic architecture (Hyde et al. 2016; International League Against Epilepsy Consortium on Complex Epilepsies 2014). PGBD1, expressed in the brain and implicated in Alzheimer's disease, highlights potential epigenetic mechanisms, though evidence for epilepsy remains limited (Bertram and Tanzi 2009; Schjeide et al. 2009). ZKSCAN3, ZKSCAN4, and ZSCAN23 belong to zinc‐finger transcription factor families associated with transcriptional regulation, autophagy, and senescence, with emerging links to neurodegeneration and cancer (Wu et al. 2022; Hu et al. 2020; Zhong and Zhong 2021). Collectively, these findings suggest that pleiotropic genes identified here are biologically plausible candidates mediating the overlap between epilepsy and psychiatric disorders, underscoring the relevance of our genetic results.

Functional enrichment highlighted the MAPK/ERK signaling pathway, which is known to regulate cellular growth, differentiation, and synaptic excitability and has been implicated in multiple neuropsychiatric conditions (Volmat and Pouyssegur 2001; Wen et al. 2016; Liu et al. 2017; Dwivedi et al. 2006; Dwivedi et al. 2009). Dysregulation of MAPK signaling has been observed in epilepsy models as well as in depression and other psychiatric disorders, suggesting it may represent a shared biological mechanism underlying comorbidity. As for causality, prior epidemiological studies have reported bidirectional associations between epilepsy and ADHD as well as increased risk of MDD in epilepsy patients, while BIP also frequently co‐occurs with epilepsy (Knott et al. 2015; Forty et al. 2014; Kwon and Park 2014). Our bidirectional Mendelian randomization analysis provided a novel causal inference perspective on the complex associations between epilepsy and psychiatric disorders. The results indicated no significant causal effects for the majority of tested psychiatric disorders; however, we obtained evidence consistent with potential causal roles for specific traits: genetic predisposition to ADHD and MDD was associated with an increased risk of epilepsy, whereas genetic liability to BIP was associated with a decreased risk. These specific associations are supported by external evidence. The positive associations of ADHD and MDD with epilepsy align with extensive epidemiological studies documenting their high comorbidity (Ahlqvist et al. 2024; Chu et al. 2024) and may be explained by shared genetic architecture. The inverse association with BIP also finds precedent in certain clinical observations (Li et al. 2023), suggesting the relationship may be modulated by specific subtypes or treatment factors. Critically, the overall pattern–where significant MR signals were the exception rather than the rule–provides a key to interpreting findings from our other analyses. It implies that the broad genetic correlations revealed by LD score regression and the shared loci identified by cross‐trait association analysis are more fundamentally attributable to widespread genetic pleiotropy. Therefore, for most psychiatric disorders, their epidemiological comorbidity with epilepsy is more likely to arise from this shared genetic foundation rather than unidirectional causal mechanisms. In summary, our multi‐method study delineates a layered landscape: widespread genetic sharing and locus pleiotropy form the primary background, while for a subset of disorders such as ADHD, MDD, and BIP, genetic liability may play a more direct causal role.

To the best of our knowledge, this is the first study to investigate gene‐level pleiotropy between psychiatric disorders and epilepsy. Nonetheless, several limitations should be acknowledged. First, although our findings suggest shared genetic components underlying these comorbidities, the functional roles of the identified pleiotropic genes remain unclear. Importantly, the effects of these genetic variants are likely modulated by environmental factors and gene‐environment interactions, which our study was not designed to capture. For instance, early‐life stress, trauma, or specific medications could interact with genetic liability to influence the ultimate phenotypic expression of either epilepsy or psychiatric disorders. Further experimental and methodological studies are required to clarify their biological significance in context. Second, our analyses relied primarily on GWAS data from individuals of European ancestry, limiting the generalizability of our findings to other populations. Differences in allele frequencies, linkage disequilibrium, epidemiology, social and cultural environments, and epidemiology across ancestries may alter genetic correlations and pleiotropic signals. Critically, the environmental exposures that potentially interact with genetic risk (e.g., infectious burden, nutritional factors, healthcare access) vary considerably across global populations, further complicating cross‐ancestry extrapolation. Third, there was a marked imbalance in GWAS sample sizes across disorders (ranging from 9,725 for OCD to 807,553 for MDD), which may have reduced statistical power for conditions with smaller datasets. Larger and more balanced samples will be essential to validate these results. Finally, our analyses used an “all‐epilepsies” GWAS phenotype combining focal and generalized forms, which prevents subtype‐specific analyses. Such heterogeneity may obscure or distort true genetic correlations with psychiatric disorders (e.g., schizophrenia) and partly explain the small negative estimate observed. Future work should apply subtype‐resolved GWAS, cross‐ancestry replication, and fine‐mapping to clarify shared versus subtype‐specific architecture. Taken together, these limitations indicate that our results should be interpreted with caution and highlight the need for larger, more diverse studies that integrate environmental measures to elucidate the complex interplay of genetic and non‐genetic factors in the future.

Based on this study, future work should focus on three priorities to deepen mechanistic understanding: First, conduct subtype‐specific analyses to validate genetic associations within precisely defined epilepsy subtypes, distinguishing shared from distinct biological bases. Second, systematically quantify environmental influences by measuring in large cohorts how factors like trauma or infection modulate the identified shared genetic risk. Third, advance experimental validation through cross‐ancestry fine‐mapping and functional studies in cellular/animal models for key targets like VRK2 and the MAPK pathway. This work will provide a substantive foundation for the precise prevention and treatment of these comorbidities.

In conclusion, our results provide evidence of shared pathogenic mechanisms between epilepsy and SCZ, ADHD, and MDD. Our findings provide new insights into the genetic overlap between epilepsy and mental disorders and are beneficial for a better understanding of the pathogenesis of epilepsy.

## Author Contributions

**Xia Feng**: Conceptualization, Visualization, Writing – original draft. **Huan Yao, Gui Xiao**: Methodolog, Data curation, Validation, Writing – review & editing. **Gui Xiao**: Supervision, Formal analysis, Project administration, Funding acquisition. All authors read and approved the final manuscript.

## Funding

This work was supported by the Natural Science Foundation of Jiangxi Province (Grant No. 20252BAC200567) and the Research Initiation Grant for PhDs of Jiangxi University of Chinese Medicine (Grant No. 2025BSZR004) Key Advantageous Discipline Construction Project of Guizhou Provincial Health Commission in 2025.

## Supporting information




**Figure S1** MAGMA analysis of the enrichment of pleiotropic loci between 14 psychiatric disorders and epilepsy. (A) Alzheimer's disease and epilepsy; (B) major depressive disorder and epilepsy; (C) Tourette syndrome and epilepsy; (D) cannabis use disorder and epilepsy; (E) anxiety disorder and epilepsy; (F) attention deficit hyperactivity disorder and epilepsy; (G) obsessive‐compulsive disorder and epilepsy; (H) autism spectrum disorder and epilepsy; (I) AUDIT‐D and epilepsy; (J) AUDIT‐C and epilepsy; (K) AUDIT‐P and epilepsy; (L) schizophrenia and epilepsy; (M) bipolar disorder and epilepsy; (N) Post‐traumatic stress disorder and epilepsy.

**Figure S2** Pleiotropic analysis and QQ plot between 14 psychiatric disorders and epilepsy. (A) Alzheimer's disease and epilepsy; (B) major depressive disorder and epilepsy; (C) Tourette syndrome and epilepsy; (D) cannabis use disorder and epilepsy; (E) anxiety disorder and epilepsy; (F) attention deficit hyperactivity disorder and epilepsy; (G) obsessive‐compulsive disorder and epilepsy; (H) autism spectrum disorder and epilepsy; (I) AUDIT‐D and epilepsy; (J) AUDIT‐C and epilepsy; (K) AUDIT‐P and epilepsy; (L) schizophrenia and epilepsy; (M) bipolar disorder and epilepsy; (N) post‐traumatic stress disorder and epilepsy.

**Figure S3** Expression of six pleiotropic genes in GTEx v8 54 tumor tissues

**Figure S4** Enrichment of six pleiotropic genes in GTEx v8 54 species of tumor tissues

**Figure S5** Scatter plots and funnel plots with a causal correlation. (A) scatter plots of genetic associations between epilepsy and attention deficit hyperactivity disorder; (B) scatter plots of genetic associations between epilepsy and bipolar disorder; (C) scatter plots of genetic associations between epilepsy and major depressive disorder; (D) funnel plots of genetic associations between epilepsy and attention deficit hyperactivity disorder; (E) funnel plots of genetic associations between epilepsy and bipolar disorder; (F) funnel plots of genetic associations between epilepsy and major depressive disorder.

**Supplementary Materials**: brb371267‐sup‐0006‐SuppMat.docx

**Table S1** The formula used to calculate R^2^ and F statistics for instrumental variables.
**Table S2** The detailed results of LDSC.
**Table S3** The detailed results of index SNPs.
**Table S4** Results of significant causal correlations between epilepsy and psychiatric disorders.
**Table S5** Results of sensitivity analyses for significant causal correlations between epilepsy and psychiatric disorders.

## Data Availability

The data that support the findings of this study are available in the International League Against Epilepsy (ILAE) at https://www.epigad.org/gwas_ilae2018_16loci.html. These data were derived from the following resources available in the public domain: ‐ Epilepsy GWAS summary statistics, https://www.med.unc.edu/pgc/download‐results/scz.

## References

[brb371267-bib-0001] Ahlqvist, V. H. , C. Dardani , P. Madley‐Dowd , et al. 2024. “Psychiatric Comorbidities in Epilepsy: Population Co‐Occurrence, Genetic Correlations and Causal Effects.” General Psychiatry 37, no. 1: e101201. 39228867 10.1136/gpsych-2023-101201 PMC11369844

[brb371267-bib-0002] Albert‐Gascó, H. , F. Ros‐Bernal , E. Castillo‐Gómez , and F. E. Olucha‐Bordonau . 2020. “MAP/ERK Signaling in Developing Cognitive and Emotional Function and Its Effect on Pathological and Neurodegenerative Processes.” International Journal of Molecular Sciences 21, no. 12: 4471. 32586047 10.3390/ijms21124471 PMC7352860

[brb371267-bib-0003] Asadi‐Pooya, A. A. , D. Dlugos , C. Skidmore , and M. R. Sperling . 2017. “Atlas of Electroencephalography, 3rd Edition.” Epileptic Disorders 19, no. 3: 384. 28872032 10.1684/epd.2017.0934

[brb371267-bib-0004] Genomes Project Consortium . et al. 2015. “A Global Reference for human Genetic Variation.” Nature 526, no. 7571: 68–74. 26432245 10.1038/nature15393 PMC4750478

[brb371267-bib-0005] Bertram, L. , and R. E Tanzi . 2009. “Genome‐Wide Association Studies in Alzheimer's Disease.” Human Molecular Genetics 18, no. R2: R137–R145. 19808789 10.1093/hmg/ddp406 PMC2758713

[brb371267-bib-0006] Bowden, J. , G. Davey Smith , P. C. Haycock , and S. Burgess . 2016. “Consistent Estimation in Mendelian Randomization With Some Invalid Instruments Using a Weighted Median Estimator.” Genetic Epidemiology 40, no. 4: 304–314. 27061298 10.1002/gepi.21965 PMC4849733

[brb371267-bib-0007] Bowden, J. , M. F. Del Greco , C. Minelli , G. Davey Smith , N. A. Sheehan , and J. R. Thompson . 2016. “Assessing the Suitability of Summary Data for Two‐Sample Mendelian Randomization Analyses Using MR‐Egger Regression: the Role of the I2 Statistic.” International Journal of Epidemiology 45, no. 6: 1961–1974. 27616674 10.1093/ije/dyw220 PMC5446088

[brb371267-bib-0008] Bozarth, X. , J. N. Dines , Q. Cong , et al. 2018. “Expanding Clinical Phenotype in CACNA1C Related Disorders: From Neonatal Onset Severe Epileptic Encephalopathy to Late‐onset Epilepsy.” American Journal of Medical Genetics Part A 176, no. 12: 2733–2739. 30513141 10.1002/ajmg.a.40657 PMC6312477

[brb371267-bib-0009] Brunklaus, A. , T. Brunger , T. Feng , et al. 2022. “The Gain of Function SCN1A Disorder Spectrum: Novel Epilepsy Phenotypes and Therapeutic Implications.” Brain 145, no. 11: 3816–3831. 35696452 10.1093/brain/awac210 PMC9679167

[brb371267-bib-0010] Bulik‐Sullivan, B. , H. K. Finucane , V. Anttila , et al. 2015. “An Atlas of Genetic Correlations Across human Diseases and Traits.” Nature Genetics 47, no. 11: 1236–1241. 26414676 10.1038/ng.3406 PMC4797329

[brb371267-bib-0011] Bulik‐Sullivan, B. K. , P. R. Loh , H. K. Finucane , et al. 2015. “LD Score Regression Distinguishes Confounding From Polygenicity in Genome‐Wide Association Studies.” Nature Genetics 47, no. 3: 291–295. 25642630 10.1038/ng.3211 PMC4495769

[brb371267-bib-0012] Burgess, S. , D. S. Small , and S. G. Thompson . 2017. “A Review of Instrumental Variable Estimators for Mendelian Randomization.” Statistical Methods in Medical Research 26, no. 5: 2333–2355. 26282889 10.1177/0962280215597579 PMC5642006

[brb371267-bib-0013] Burgess, S. , and S. G. Thompson . 2017. “Interpreting Findings From Mendelian Randomization Using the MR‐Egger Method.” European Journal of Epidemiology 32, no. 5: 377–389. 28527048 10.1007/s10654-017-0255-x PMC5506233

[brb371267-bib-0014] Campbell, C. , G. L. Cavalleri , and N. Delanty . 2020. “Exploring the Genetic Overlap Between Psychiatric Illness and Epilepsy: a Review.” Epilepsy & Behavior 102: 106669. 31785486 10.1016/j.yebeh.2019.106669

[brb371267-bib-0015] Cascella, N. G. , D. J. Schretlen , and A. Sawa . 2009. “Schizophrenia and Epilepsy: Is There a Shared Susceptibility?” Neuroscience Research 63, no. 4: 227–235. 19367784 10.1016/j.neures.2009.01.002 PMC2768382

[brb371267-bib-0016] Chou, I. C. , Y. T. Chang , Z. N. Chin , et al. 2013. “Correlation Between Epilepsy and Attention Deficit Hyperactivity Disorder: a Population‐Based Cohort Study.” PLoS ONE 8, no. 3:e57926. 23483944 10.1371/journal.pone.0057926 PMC3590288

[brb371267-bib-0017] Chu, H. , B. Wang , X. Zhao , and L. Mu . 2024. “Epilepsy and Psychiatric Comorbidities: A Bidirectional Mendelian Randomization Study.” Journal of Affective Disorders 350: 774–783. 38272360 10.1016/j.jad.2024.01.178

[brb371267-bib-0018] Cunningham, F. , M. R. Amode , D. Barrell , et al. 2015. “Ensembl 2015.” Nucleic Acids Research 43: D662–D669. 25352552 10.1093/nar/gku1010 PMC4383879

[brb371267-bib-0019] de Leeuw, C. A. , J. M. Mooij , T. Heskes , and D. Posthuma . 2015. “MAGMA: Generalized Gene‐Set Analysis of GWAS Data.” Plos Computational Biology 11, no. 4:e1004219. 25885710 10.1371/journal.pcbi.1004219 PMC4401657

[brb371267-bib-0020] Demontis, D. , R. K. Walters , J. Martin , et al. 2019. “Discovery of the First Genome‐wide Significant Risk Loci for Attention Deficit/Hyperactivity Disorder.” Nature Genetics 51, no. 1: 63–75. 30478444 10.1038/s41588-018-0269-7 PMC6481311

[brb371267-bib-0021] Duncan, J. S. , J. W. Sander , S. M. Sisodiya , and M. C Walker . 2006. “Adult Epilepsy.” Lancet 367, no. 9516: 1087–1100. 16581409 10.1016/S0140-6736(06)68477-8

[brb371267-bib-0022] Duncan, L. , Z. Yilmaz , H. Gaspar , et al. 2017. “Significant Locus and Metabolic Genetic Correlations Revealed in Genome‐Wide Association Study of Anorexia Nervosa.” American Journal of Psychiatry 174, no. 9: 850–858. 28494655 10.1176/appi.ajp.2017.16121402 PMC5581217

[brb371267-bib-0023] Dunn, D. W. , J. K. Austin , J. Harezlak , and W. T Ambrosius . 2003. “ADHD and Epilepsy in Childhood.” Developmental Medicine and Child Neurology 45, no. 1: 50–54. 12549755

[brb371267-bib-0024] Dwivedi, Y. , H. S. Rizavi , R. R. Conley , and G. N. Pandey . 2006. “ERK MAP Kinase Signaling in Post‐Mortem Brain of Suicide Subjects: Differential Regulation of Upstream Raf Kinases Raf‐1 and B‐Raf.” Molecular Psychiatry 11, no. 1: 86–98. 16172610 10.1038/sj.mp.4001744

[brb371267-bib-0025] Dwivedi, Y. , H. S. Rizavi , H. Zhang , R. C. Roberts , R. R. Conley , and G. N. Pandey . 2009. “Aberrant Extracellular Signal‐Regulated Kinase (ERK)1/2 Signalling in Suicide Brain: Role of ERK Kinase 1 (MEK1).” The International Journal of Neuropsychopharmacology 12, no. 10: 1337–1354. 19835659 10.1017/S1461145709990575

[brb371267-bib-0026] Escayg, A. , B. T. MacDonald , and M. H. Meisler , et al. 2000. “Mutations of SCN1A, Encoding a Neuronal Sodium Channel, in Two Families With GEFS+2.” Nature Genetics 24, no. 4: 343–345. 10742094 10.1038/74159

[brb371267-bib-0027] Forty, L. , A. Ulanova , and L. Jones , et al. 2014. “Comorbid Medical Illness in Bipolar Disorder.” British Journal of Psychiatry 205, no. 6: 465–472. 10.1192/bjp.bp.114.152249 PMC4248234 25359927

[brb371267-bib-0028] Gaitatzis, A. , K. Carroll , A. Majeed , and J. W. Sander . 2004. “The Epidemiology of the Comorbidity of Epilepsy in the General Population.” Epilepsia 45, no. 12: 1613–1622. 15571520 10.1111/j.0013-9580.2004.17504.x

[brb371267-bib-0029] Grove, J. , S. Ripke , T. D. Als , et al. 2019. “Identification of Common Genetic Risk Variants for Autism Spectrum Disorder.” Nature Genetics 51, no. 3: 431–444. 30804558 10.1038/s41588-019-0344-8 PMC6454898

[brb371267-bib-0030] Hollis, A. , and J. R. Lukens . 2025. “Role of Inflammasomes and Neuroinflammation in Epilepsy.” Immunological Reviews 329, no. 1:e13421. 39523682 10.1111/imr.13421 PMC11744240

[brb371267-bib-0031] Howard, D. M. , M. J. Adams , T. K. Clarke , et al. 2019. “Genome‐Wide Meta‐Analysis of Depression Identifies 102 Independent Variants and Highlights the Importance of the Prefrontal Brain Regions.” Nature Neuroscience 22, no. 3: 343–352. 30718901 10.1038/s41593-018-0326-7 PMC6522363

[brb371267-bib-0032] Hu, H. , Q. Ji , and M. Song , et al. 2020. “ZKSCAN3 counteracts Cellular Senescence by Stabilizing Heterochromatin.” Nucleic Acids Research 48, no. 11: 6001–6018. 32427330 10.1093/nar/gkaa425 PMC7293006

[brb371267-bib-0033] Hyde, C. L. , M. W. Nagle , C. Tian , et al. 2016. “Identification of 15 Genetic Loci Associated With Risk of Major Depression in Individuals of European Descent.” Nature Genetics 48, no. 9: 1031–1036. 27479909 10.1038/ng.3623 PMC5706769

[brb371267-bib-0034] International League Against Epilepsy Consortium on Complex E . 2018. “Genome‐Wide Mega‐Analysis Identifies 16 Loci and Highlights Diverse Biological Mechanisms in the Common Epilepsies.” Nature Communications 9, no. 1: 5269. 10.1038/s41467-018-07524-z PMC6288131 30531953

[brb371267-bib-0035] International League Against Epilepsy Consortium on Complex Epilepsies . 2014. “Electronic Address E‐auea. Genetic Determinants of Common Epilepsies: A Meta‐Analysis of Genome‐Wide Association Studies.” Lancet Neurology 13, no. 9: 893–903. 25087078 10.1016/S1474-4422(14)70171-1 PMC4189926

[brb371267-bib-0036] International Obsessive Compulsive Disorder Foundation Genetics C, Studies OCDCGA . 2018. “Revealing the Complex Genetic Architecture of Obsessive‐compulsive Disorder Using Meta‐Analysis.” Molecular Psychiatry 23, no. 5: 1181–1188. 28761083 10.1038/mp.2017.154 PMC6660151

[brb371267-bib-0037] Jansen, I. E. , J. E. Savage , K. Watanabe , et al. 2019. “Genome‐Wide Meta‐Analysis Identifies New Loci and Functional Pathways Influencing Alzheimer's Disease Risk.” Nature Genetics 51, no. 3: 404–413. 30617256 10.1038/s41588-018-0311-9 PMC6836675

[brb371267-bib-0038] Johnson, E. C. , D. Demontis , T. E. Thorgeirsson , et al. 2020. “A Large‐Scale Genome‐Wide Association Study Meta‐Analysis of Cannabis Use Disorder.” Lancet Psychiatry 7, no. 12: 1032–1045. 33096046 10.1016/S2215-0366(20)30339-4 PMC7674631

[brb371267-bib-0039] Kim, M. , Y. S. Kim , D. H. Kim , T. W. Yang , and O. Y. Kwon . 2018. “Major Depressive Disorder in Epilepsy Clinics: A Meta‐Analysis.” Epilepsy & Behavior 84: 56–69. 29753295 10.1016/j.yebeh.2018.04.015

[brb371267-bib-0040] Knott, S. , L. Forty , N. Craddock , and R. H. Thomas . 2015. “Epilepsy and Bipolar Disorder.” Epilepsy & Behavior 52, no. Pt A: 267–274. 26316422 10.1016/j.yebeh.2015.07.003

[brb371267-bib-0041] Kwon, O. Y. , and S. P. Park . 2014. “Depression and Anxiety in People With Epilepsy.” Journal of Clinical Neurology 10, no. 3: 175–188. 25045369 10.3988/jcn.2014.10.3.175 PMC4101093

[brb371267-bib-0042] Li, G. , M. Wang , M. Zheng , et al. 2023. “Causal Effect of Psychiatric Disorders on Epilepsy: A Two‐Sample Mendelian Randomization Study.” Brain and Behavior 13, no. 4:e2939. 36860142 10.1002/brb3.2939 PMC10097067

[brb371267-bib-0043] Liu, W. , T. Ge , and Y. Leng , et al. 2017. “The Role of Neural Plasticity in Depression: From Hippocampus to Prefrontal Cortex.” Neural Plasticity 2017: 6871089. 28246558 10.1155/2017/6871089 PMC5299163

[brb371267-bib-0044] Makikyro, T. , J. T. Karvonen , H. Hakko , et al. 1998. “Comorbidity of Hospital‐Treated Psychiatric and Physical Disorders With Special Reference to Schizophrenia: a 28 Year Follow‐Up of the 1966 northern Finland General Population Birth Cohort.” Public Health 112, no. 4: 221–228. 9724944 10.1038/sj.ph.1900455

[brb371267-bib-0045] Mohanannair Geethadevi, G. , Z. Chen , and E. Foster , et al. 2025. “Impact of Epilepsy on Productivity and Quality of Life: The Australian Epilepsy Project.” Neurology 105, no. 5:e214011. 40829103 10.1212/WNL.0000000000214011

[brb371267-bib-0046] Mullins, N. , A. J. Forstner , and K. S. O'Connell , et al. 2021. “Genome‐Wide Association Study of More Than 40,000 Bipolar Disorder Cases Provides New Insights Into the Underlying Biology.” Nature Genetics 53, no. 6: 817–829. 34002096 10.1038/s41588-021-00857-4 PMC8192451

[brb371267-bib-0047] Nievergelt, C. M. , A. X. Maihofer , T. Klengel , et al. 2019. “International Meta‐Analysis of PTSD Genome‐Wide Association Studies Identifies Sex‐ and Ancestry‐Specific Genetic Risk Loci.” Nature Communications 10, no. 1: 4558. 10.1038/s41467-019-12576-w PMC6783435 31594949

[brb371267-bib-0048] Noyce, A. J. , D. A. Kia , and G. Hemani , et al. 2017. “Estimating the Causal Influence of Body Mass Index on Risk of Parkinson Disease: A Mendelian Randomisation Study.” Plos Medicine 14, no. 6:e1002314. 28609445 10.1371/journal.pmed.1002314 PMC5469450

[brb371267-bib-0049] Pisani, F. , L. Rosa Pisani , M. A. Barbieri , J. de Leon , and E. Spina . 2023. “Optimization of Therapy in Patients With Epilepsy and Psychiatric Comorbidities: Key Points.” Current Neuropharmacology 21, no. 8: 1755–1766. 35619263 10.2174/1570159X20666220526144314 PMC10514544

[brb371267-bib-0050] Qin, P. , H. Xu , T. M. Laursen , M. Vestergaard , and P. B. Mortensen . 2005. “Risk for Schizophrenia and Schizophrenia‐Like Psychosis Among Patients With Epilepsy: Population Based Cohort Study.” BMJ 331, no. 7507: 23. 15964859 10.1136/bmj.38488.462037.8F PMC558534

[brb371267-bib-0051] Ray, D. , and N. Chatterjee . 2020. “A Powerful Method for Pleiotropic Analysis Under Composite Null Hypothesis Identifies Novel Shared Loci Between Type 2 Diabetes and Prostate Cancer.” PLoS Genetics 16, no. 12:e1009218. 33290408 10.1371/journal.pgen.1009218 PMC7748289

[brb371267-bib-0052] Ray, D. , S. Venkataraghavan , W. Zhang , et al. 2021. “Pleiotropy Method Reveals Genetic Overlap Between Orofacial Clefts at Multiple Novel Loci From GWAS of Multi‐Ethnic Trios.” PLoS Genetics 17, no. 7:e1009584. 34242216 10.1371/journal.pgen.1009584 PMC8270211

[brb371267-bib-0053] Riney, K. , A. Bogacz , E. Somerville , et al. 2022. “International League Against Epilepsy Classification and Definition of Epilepsy Syndromes With Onset at a Variable Age: Position Statement by the ILAE Task Force on Nosology and Definitions.” Epilepsia 63, no. 6: 1443–1474. 35503725 10.1111/epi.17240

[brb371267-bib-0054] Sanchez‐Roige, S. , A. A. Palmer , P. Fontanillas , S. L. Elson , 23andMe Research Team tSUDWGotPGC . and M. J. Adams , et al. 2019. “Genome‐Wide Association Study Meta‐Analysis of the Alcohol Use Disorders Identification Test (AUDIT) in Two Population‐Based Cohorts.” American Journal of Psychiatry 176, no. 2: 107–118. 30336701 10.1176/appi.ajp.2018.18040369 PMC6365681

[brb371267-bib-0055] Scheffer, I. E. , and R. Nabbout . 2019. “SCN1A‐related Phenotypes: Epilepsy and Beyond.” Epilepsia 60, no. S3: S17–S24. 31904117 10.1111/epi.16386

[brb371267-bib-0056] Schjeide, B. M. , M. B. McQueen , K. Mullin , et al. 2009. “Assessment of Alzheimer's Disease Case‐Control Associations Using Family‐Based Methods.” Neurogenetics 10, no. 1: 19–25. 18830724 10.1007/s10048-008-0151-3 PMC2841132

[brb371267-bib-0057] Sheehan, N. A. , V. Didelez , P. R. Burton , and M. D. Tobin . 2008. “Mendelian Randomisation and Causal Inference in Observational Epidemiology.” Plos Medicine 5, no. 8:e177. 18752343 10.1371/journal.pmed.0050177 PMC2522255

[brb371267-bib-0058] Socanski, D. , D. Aurlien , A. Herigstad , P. H. Thomsen , and T. K. Larsen . 2013. “Epilepsy in a Large Cohort of Children Diagnosed With Attention Deficit/Hyperactivity Disorders (ADHD).” Seizure: The Journal of the British Epilepsy Association 22, no. 8: 651–655. 10.1016/j.seizure.2013.04.021 23711613

[brb371267-bib-0059] Subramanian, A. , P. Tamayo , V. K. Mootha , et al. 2005. “Gene Set Enrichment Analysis: a Knowledge‐based Approach for Interpreting Genome‐wide Expression Profiles.” PNAS 102, no. 43: 15545–15550. 16199517 10.1073/pnas.0506580102 PMC1239896

[brb371267-bib-0060] Sudmant, P. H. , T. Rausch , E. J. Gardner , et al. 2015. “An Integrated Map of Structural Variation in 2,504 human Genomes.” Nature 526, no. 7571: 75–81. 26432246 10.1038/nature15394 PMC4617611

[brb371267-bib-0061] Thapar, A. , and M. Cooper . 2016. “Attention Deficit Hyperactivity Disorder.” Lancet 387, no. 10024: 1240–1250. 26386541 10.1016/S0140-6736(15)00238-X

[brb371267-bib-0062] Thijs, R. D. , R. Surges , T. J. O'Brien , and J. W. Sander . 2019. “Epilepsy in Adults.” Lancet 393, no. 10172: 689–701. 30686584 10.1016/S0140-6736(18)32596-0

[brb371267-bib-0063] Tian, N. , M. Boring , R. Kobau , M. M. Zack , and J. B. Croft . 2018. “Active Epilepsy and Seizure Control in Adults—United States, 2013 and 2015.” Mmwr Morbidity and Mortality Weekly Report 67, no. 15: 437–442. 29672474 10.15585/mmwr.mm6715a1 PMC6191103

[brb371267-bib-0064] Trubetskoy, V. , A. F. Pardinas , T. Qi , et al. 2022. “Mapping Genomic Loci Implicates Genes and Synaptic Biology in Schizophrenia.” Nature 604, no. 7906: 502–508. 35396580 10.1038/s41586-022-04434-5 PMC9392466

[brb371267-bib-0065] Verbanck, M. , C. Y. Chen , B. Neale , and R. Do . 2018. “Detection of Widespread Horizontal Pleiotropy in Causal Relationships Inferred From Mendelian Randomization Between Complex Traits and Diseases.” Nature Genetics 50, no. 5: 693–698. 29686387 10.1038/s41588-018-0099-7 PMC6083837

[brb371267-bib-0066] Volmat, V. , and J. Pouyssegur . 2001. “Spatiotemporal Regulation of the p42/p44 MAPK Pathway.” Biologie Cellulaire 93, no. 1‐2: 71–79. 10.1016/s0248-4900(01)01129-7 11730325

[brb371267-bib-0067] Watanabe, K. , E. Taskesen , A. van Bochoven , and D Posthuma . 2017. “Functional Mapping and Annotation of Genetic Associations With FUMA.” Nature Communications 8, no. 1: 1826. 10.1038/s41467-017-01261-5 PMC5705698 29184056

[brb371267-bib-0068] Wen, Y. , M. J. Alshikho , and M. R. Herbert . 2016. “Pathway Network Analyses for Autism Reveal Multisystem Involvement, Major Overlaps With Other Diseases and Convergence Upon MAPK and Calcium Signaling.” PLoS ONE 11, no. 4:e0153329. 27055244 10.1371/journal.pone.0153329 PMC4824422

[brb371267-bib-0069] Witkin, J. M. , H. Shafique , R. Cerne , et al. 2024. “Mechanistic and Therapeutic Relationships of Traumatic Brain Injury and γ‐amino‐butyric Acid (GABA).” Pharmacology & Therapeutics 256: 108609. 38369062 10.1016/j.pharmthera.2024.108609

[brb371267-bib-0070] Wu, X. , Y. Ren , Y. Wen , et al. 2022. “Deacetylation of ZKSCAN3 by SIRT1 Induces Autophagy and Protects SN4741 Cells Against MPP(+)‐induced Oxidative Stress.” Free Radical Biology and Medicine 181: 82–97. 35124181 10.1016/j.freeradbiomed.2022.02.001

[brb371267-bib-0071] Yu, D. , J. H. Sul , F. Tsetsos , et al. 2019. “Interrogating the Genetic Determinants of Tourette's Syndrome and Other Tic Disorders through Genome‐Wide Association Studies.” American Journal of Psychiatry 176, no. 3: 217–227. 30818990 10.1176/appi.ajp.2018.18070857 PMC6677250

[brb371267-bib-0072] Zeng, P. , Z. Shao , and X. Zhou . 2021. “Statistical Methods for Mediation Analysis in the Era of High‐Throughput Genomics: Current Successes and Future Challenges.” Computational and Structural Biotechnology Journal 19: 3209–3224. 34141140 10.1016/j.csbj.2021.05.042 PMC8187160

[brb371267-bib-0073] Zhong, X. , and G. Zhong . 2021. “Prognostic Biomarker Identification and Tumor Classification in Breast Cancer Patients by Methylation and Transcriptome Analysis.” FEBS Open Bio 11, no. 8: 2139–2151. 10.1002/2211-5463.13211 PMC8329782 34056873

