# Supplementary figures and images for: Shared Genetic Basis and Causality Between Epilepsy and Psychiatric Disorders: Evidence From a Comprehensive Genetic Analysis
Source: Brain Behav. 2026 Feb 24;16(2):e71267. doi: 10.1002/brb3.71267 (PMC12931490; doi:10.1002/brb3.71267)

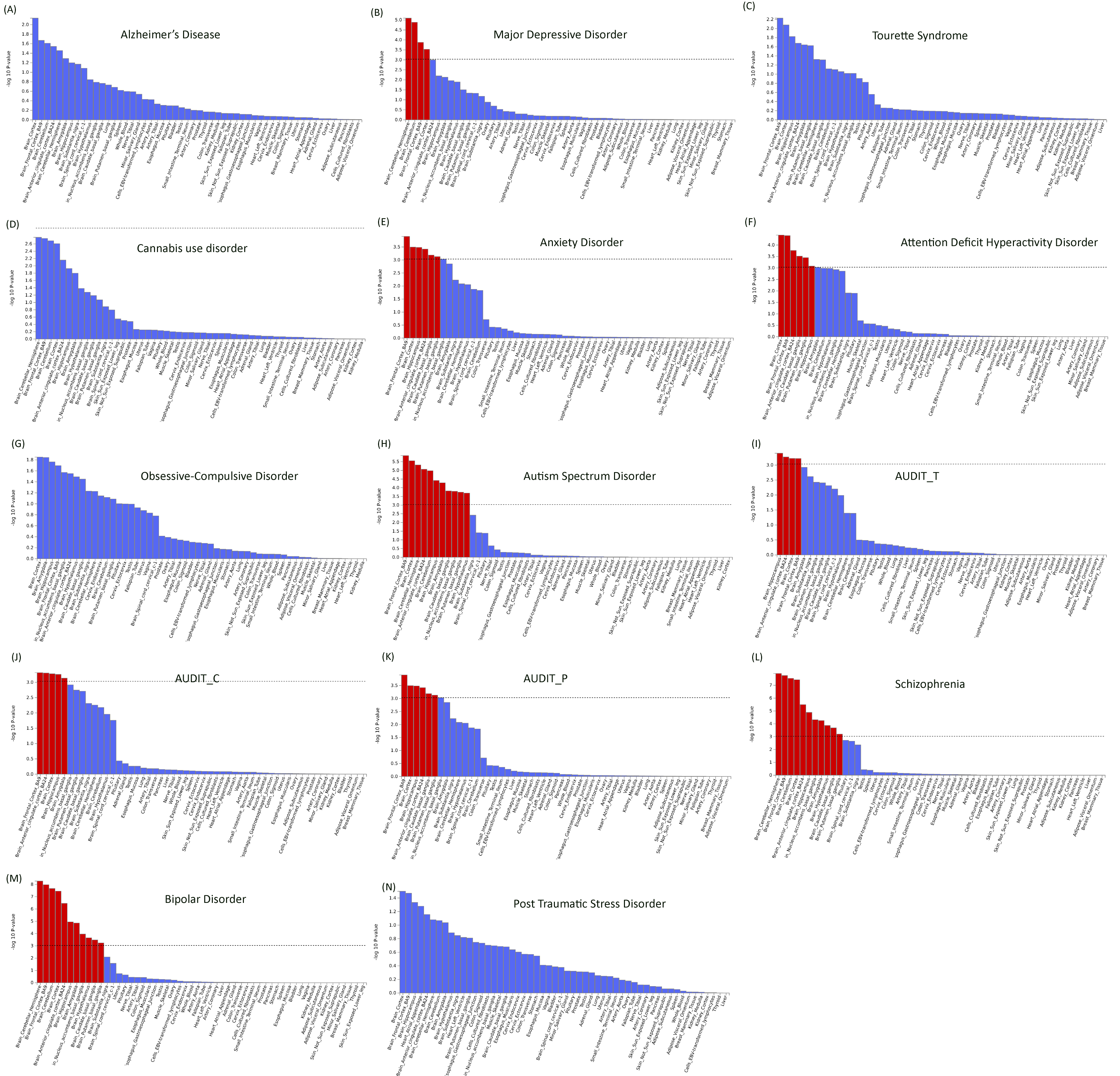

Supplement: Supplementary file 1 — Figure S1 MAGMA analysis of the enrichment of pleiotropic loci between 14 psychiatric disorders and epilepsy. (A) Alzheimer's disease and epilepsy; (B) major depressive disorder and epilepsy; (C) Tourette syndrome and epilepsy; (D) cannabis use disorder and epilepsy; (E) anxiety disorder and epilepsy; (F) attention deficit hyperactivity disorder and epilepsy; (G) obsessive‐compulsive disorder and epilepsy; (H) autism spectrum disorder and epilepsy; (I) AUDIT‐D and epilepsy; (J) AUDIT‐C and epilepsy; (K) AUDIT‐P and epilepsy; (L) schizophrenia and epilepsy; (M) bipolar disorder and epilepsy; (N) Post‐traumatic stress disorder and epilepsy. [file BRB3-16-e71267-s003.tif]

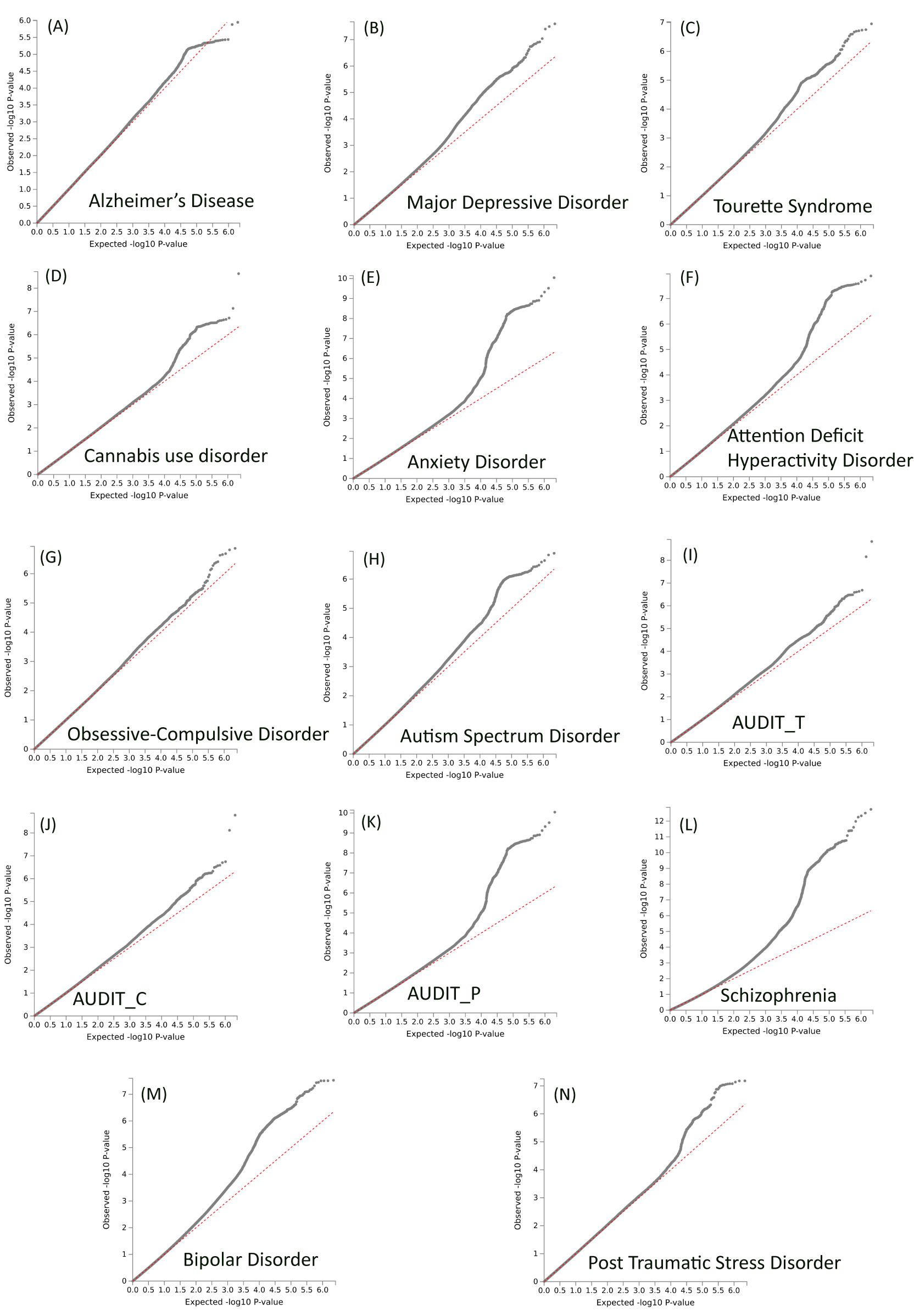

Supplement: Supplementary file 2 — Figure S2 Pleiotropic analysis and QQ plot between 14 psychiatric disorders and epilepsy. (A) Alzheimer's disease and epilepsy; (B) major depressive disorder and epilepsy; (C) Tourette syndrome and epilepsy; (D) cannabis use disorder and epilepsy; (E) anxiety disorder and epilepsy; (F) attention deficit hyperactivity disorder and epilepsy; (G) obsessive‐compulsive disorder and epilepsy; (H) autism spectrum disorder and epilepsy; (I) AUDIT‐D and epilepsy; (J) AUDIT‐C and epilepsy; (K) AUDIT‐P and epilepsy; (L) schizophrenia and epilepsy; (M) bipolar disorder and epilepsy; (N) post‐traumatic stress disorder and epilepsy. [file BRB3-16-e71267-s006.tif]

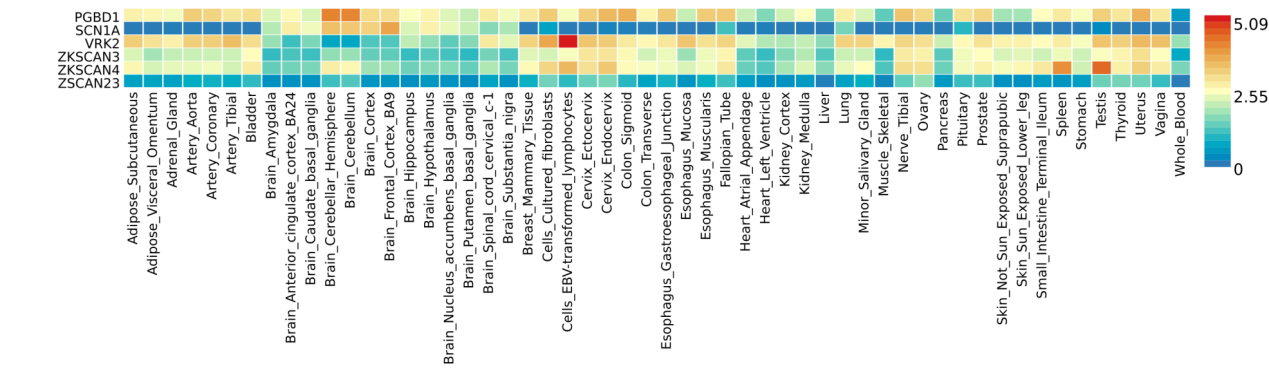

Supplement: Supplementary file 3 — Figure S3 Expression of six pleiotropic genes in GTEx v8 54 tumor tissues [file BRB3-16-e71267-s002.tif]

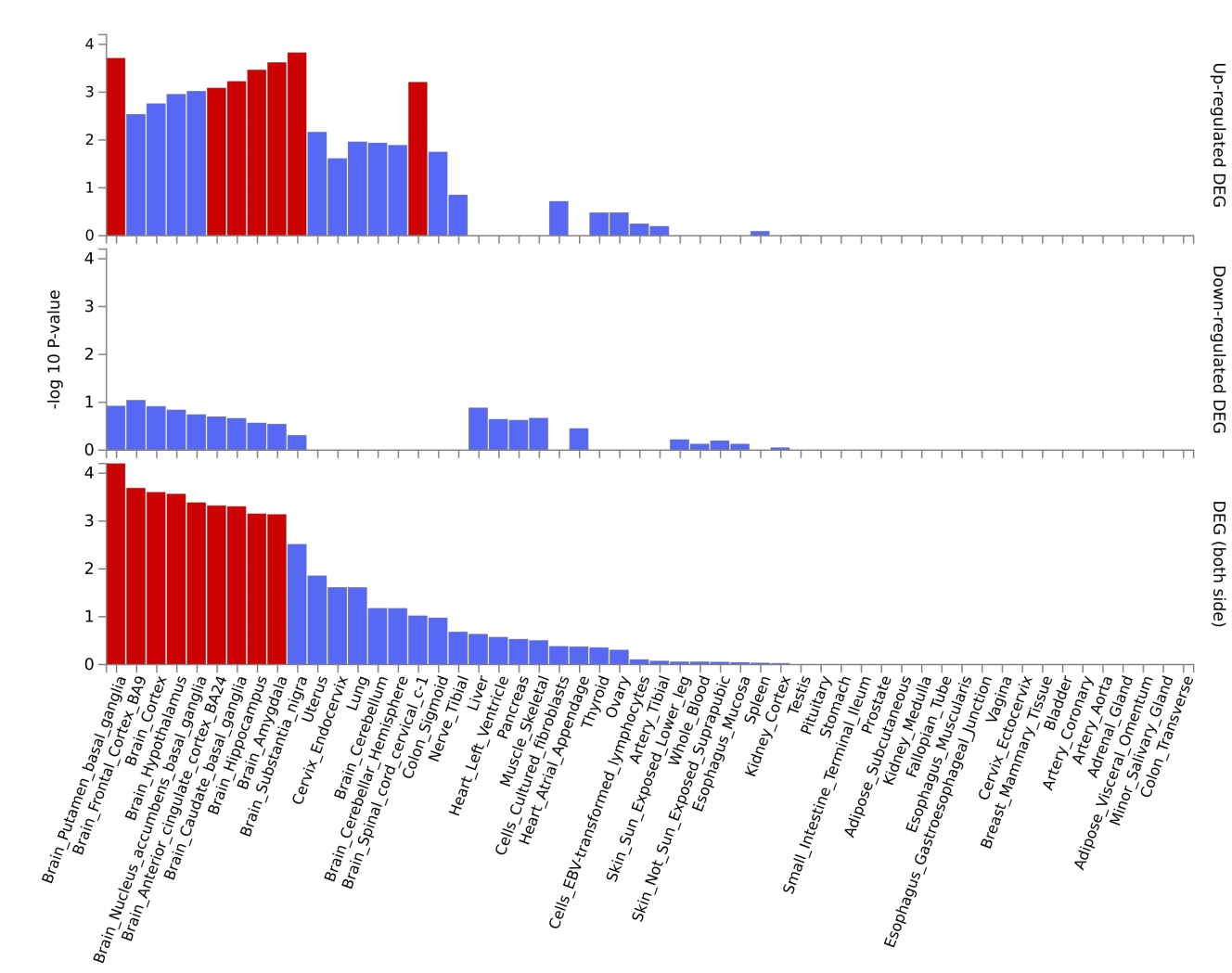

Supplement: Supplementary file 4 — Figure S4 Enrichment of six pleiotropic genes in GTEx v8 54 species of tumor tissues [file BRB3-16-e71267-s005.tif]

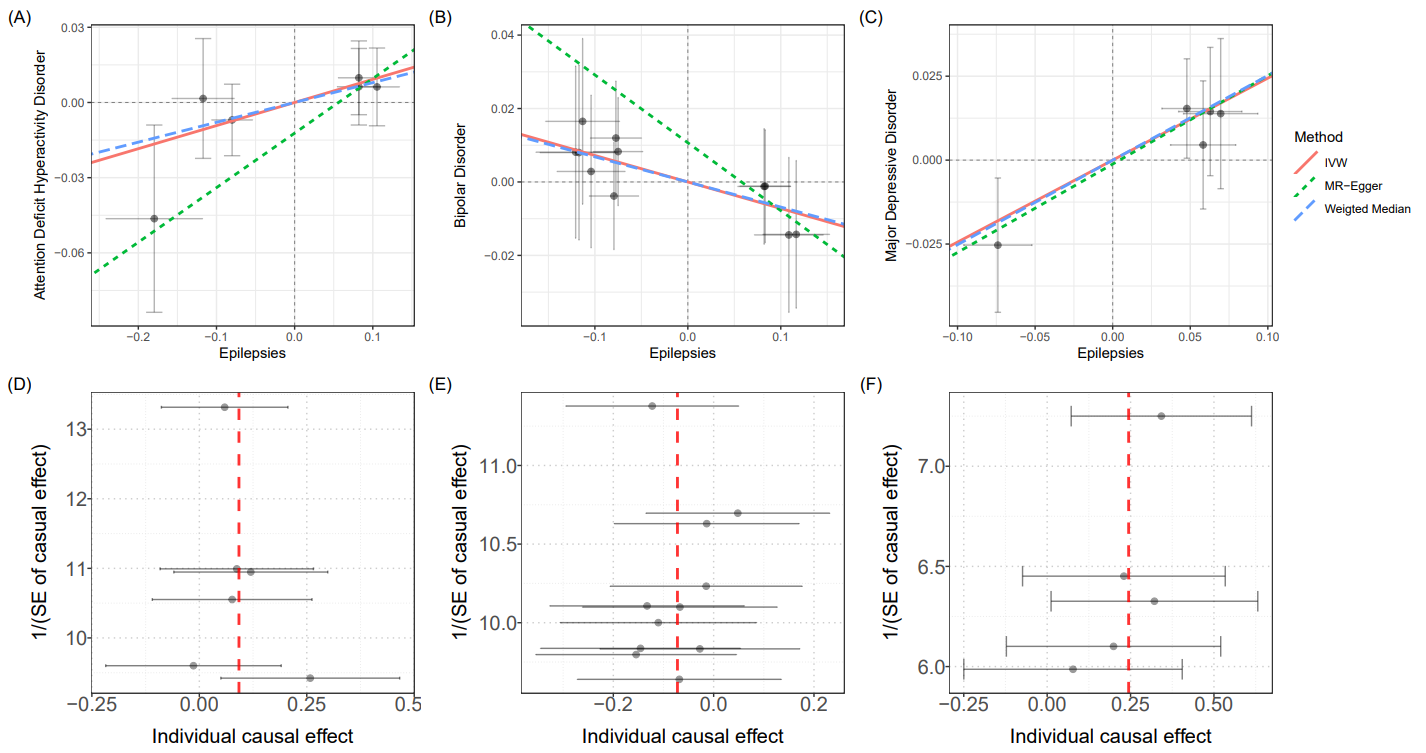

Supplement: Supplementary file 5 — Figure S5 Scatter plots and funnel plots with a causal correlation. (A) scatter plots of genetic associations between epilepsy and attention deficit hyperactivity disorder; (B) scatter plots of genetic associations between epilepsy and bipolar disorder; (C) scatter plots of genetic associations between epilepsy and major depressive disorder; (D) funnel plots of genetic associations between epilepsy and attention deficit hyperactivity disorder; (E) funnel plots of genetic associations between epilepsy and bipolar disorder; (F) funnel plots of genetic associations between epilepsy and major depressive disorder. [file BRB3-16-e71267-s004.tif]
