# Supplementary material for: Shared Genetic Basis and Causality Between Epilepsy and Psychiatric Disorders: Evidence From a Comprehensive Genetic Analysis
Source: Brain Behav. 2026 Feb 24;16(2):e71267. doi: 10.1002/brb3.71267 (PMC12931490; doi:10.1002/brb3.71267)
Supplement: Supplementary file 6 — Supplementary Materials: brb371267‐sup‐0006‐SuppMat.docx [file BRB3-16-e71267-s001.docx]

**Supplementary Methods**

**2.4 Gene-based pleiotropic analysis under PLACO**

**2.4.1 SNP-level pleiotropy test (PLACO)**

To identify pleiotropic genetic variants shared between epilepsy and each psychiatric disorder, we applied the PLACO method (Pleiotropic Analysis under a Composite Null Hypothesis) (1). PLACO is a summary-statistics–based test that evaluates, for each SNP, whether its genetic effect is simultaneously non-zero on both traits. Formally, the composite null hypothesis comprises three sub-scenarios: (i) the SNP is associated with neither trait (H₀₀), (ii) it is associated only with epilepsy (H₁₀), or (iii) it is associated only with the psychiatric disorder (H₀₁). The alternative hypothesis (H₁₁) is that the SNP affects both traits, indicating pleiotropy.

In practice, PLACO takes as input the GWAS Z-scores for epilepsy and for each psychiatric disorder at the same SNP and constructs a test statistic based on their product. The null distribution of this statistic explicitly accounts for correlation between the two sets of Z-scores, which can arise from sample overlap and from genetic correlation between traits. This feature allows PLACO to retain correct type I error while being more powerful than ad hoc overlap-sensitive approaches, particularly for variants whose single-trait effects are modest but consistent in direction across traits.

For PLACO, we started from the quality-controlled GWAS summary statistics provided by the original consortia and further restricted the analysis to autosomal SNPs present in both the epilepsy and psychiatric-disorder GWAS. We aligned effect and non-effect alleles to ensure consistent coding of the risk allele across traits, excluded strand-ambiguous SNPs (A/T and C/G) with high minor allele frequency, and removed variants within the extended major histocompatibility complex (MHC) region (chr6: ~25–34 Mb) because of its complex linkage disequilibrium structure. In addition, we required minor allele frequency ≥ 0.01 and imputation quality (INFO or equivalent metric) ≥ 0.80 in both GWAS datasets. To avoid undue influence from extreme outliers, we calculated the squared Z-score for each variant and excluded SNPs with Z^2^>80. Following the original PLACO implementation, we estimated the cross-trait correlation matrix of the Z-scores to correctly account for sample overlap and residual dependence between the epilepsy and psychiatric-disorder GWAS. PLACO p values were then obtained for each SNP, and variants reaching genome-wide significance (PLACO p<5 × 10^-8^) were considered pleiotropic loci and prioritized for downstream annotation and functional follow-up (2).

**2.4.2 Gene-level pleiotropy analysis using MAGMA**

To aggregate SNP-level pleiotropic signals into more interpretable gene-level associations, we performed gene-based analysis with MAGMA (v1.07b) (3). MAGMA implements an annotation-based multi-marker test that combines SNP-level association statistics within a gene while accounting for local linkage disequilibrium, yielding a single gene-level test statistic. In this study, we used the PLACO-derived SNP-level statistics as input, so that the resulting gene-level signals reflect pleiotropic effects shared between epilepsy and the psychiatric disorder.

We restricted the analysis to 18,563 autosomal protein-coding genes. SNPs were mapped to genes using an annotation window of ±500 kb around the transcription start and end sites, thereby capturing nearby regulatory variants that may influence gene function. Gene coordinates were obtained from Ensembl (GRCh37 build) (4), and linkage disequilibrium information was derived from the European subset of the 1000 Genomes Project reference panel. MAGMA’s SNP-wise multi-marker model was then used to calculate a gene-level p value summarizing the joint contribution of all SNPs assigned to each gene.

To control for multiple testing, both PLACO SNP-level p values and MAGMA gene-level p values were corrected using Bonferroni adjustment across the number of SNPs and genes tested, respectively. Genes surpassing the Bonferroni-corrected threshold were considered candidate pleiotropic genes. These genes were taken forward into downstream functional annotation and pathway analyses, providing a bridge from variant-level pleiotropic signals to biologically interpretable mechanisms shared by epilepsy and psychiatric disorders.

**Reference**

1. Ray D, Chatterjee N. A powerful method for pleiotropic analysis under composite null hypothesis identifies novel shared loci between Type 2 Diabetes and Prostate Cancer. PLoS Genet. 2020;16(12):e1009218.

2. Noyce AJ, Kia DA, Hemani G, Nicolas A, Price TR, De Pablo-Fernandez E, et al. Estimating the causal influence of body mass index on risk of Parkinson disease: A Mendelian randomisation study. PLoS Med. 2017;14(6):e1002314.

3. de Leeuw CA, Mooij JM, Heskes T, Posthuma D. MAGMA: generalized gene-set analysis of GWAS data. PLoS Comput Biol. 2015;11(4):e1004219.

4. Cunningham F, Amode MR, Barrell D, Beal K, Billis K, Brent S, et al. Ensembl 2015. Nucleic Acids Res. 2015;43(Database issue):D662-9.
